# HOXA‐AS2 Epigenetically Inhibits HBV Transcription by Recruiting the MTA1‐HDAC1/2 Deacetylase Complex to cccDNA Minichromosome

**DOI:** 10.1002/advs.202306810

**Published:** 2024-04-22

**Authors:** YiPing Qin, JiHua Ren, HaiBo Yu, Xin He, ShengTao Cheng, WeiXian Chen, Zhen Yang, FengMing Sun, ChunDuo Wang, SiYu Yuan, Peng Chen, DaiQing Wu, Fang Ren, AiLong Huang, Juan Chen

**Affiliations:** ^1^ Institute for Viral Hepatitis Key Laboratory of Molecular Biology for Infectious Diseases (Ministry of Education) Department of Infectious Diseases The Second Affiliated Hospital Chongqing Medical University Chongqing 400010 China; ^2^ Chongqing Key Laboratory of Translational Research for Cancer Metastasis and Individualized Treatment Chongqing University Cancer Hospital Chongqing 400030 China; ^3^ State Key Laboratory of Ultrasound in Medicine and Engineering College of Biomedical Engineering Chongqing Medical University Chongqing 400016 China; ^4^ Key Laboratory of Clinical Laboratory Diagnostics (Ministry of Education) College of Laboratory Medicine Chongqing Medical University Chongqing 400016 China

**Keywords:** cccDNA, Hepatitis B virus, HOXA‐AS2, MTA1‐HDAC1/2 deacetylase complex, self‐limiting HBV replication

## Abstract

Persistent transcription of HBV covalently closed circular DNA (cccDNA) is critical for chronic HBV infection. Silencing cccDNA transcription through epigenetic mechanisms offers an effective strategy to control HBV. Long non‐coding RNAs (lncRNAs), as important epigenetic regulators, have an unclear role in cccDNA transcription regulation. In this study, lncRNA sequencing (lncRNA seq) is conducted on five pairs of HBV‐positive and HBV‐negative liver tissue. Through analysis, HOXA‐AS2 (HOXA cluster antisense RNA 2) is identified as a significantly upregulated lncRNA in HBV‐infected livers. Further experiments demonstrate that HBV DNA polymerase (DNA pol) induces HOXA‐AS2 after establishing persistent high‐level HBV replication. Functional studies reveal that HOXA‐AS2 physically binds to cccDNA and significantly inhibits its transcription. Mechanistically, HOXA‐AS2 recruits the MTA1‐HDAC1/2 deacetylase complex to cccDNA minichromosome by physically interacting with metastasis associated 1 (MTA1) subunit, resulting in reduced acetylation of histone H3 at lysine 9 (H3K9ac) and lysine 27 (H3K27ac) associated with cccDNA and subsequently suppressing cccDNA transcription. Altogether, the study reveals a mechanism to self‐limit HBV replication, wherein the upregulation of lncRNA HOXA‐AS2, induced by HBV DNA pol, can epigenetically suppress cccDNA transcription.

## Graphical abstract

The study found that HBV DNA pol upregulates the expression of host lncRNA HOXA‐AS2 after establishing persistent high‐level HBV replication. Subsequently, HOXA‐AS2 directly binds to HBV cccDNA and recruits MTA1‐HDAC1/2 deacetylase complexes, epigenetically suppressing HBV transcription. These findings provide new insights into the epigenetic inhibition of cccDNA transcription by lncRNAs and elucidate a mechanism for self‐limiting HBV replication.

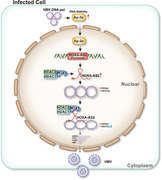

## 1 Introduction

Hepatitis B Virus (HBV) is a significant global health concern, with ≈296 million people worldwide living with chronic infections.^[^ ^1^ ^]^ Chronic HBV infection can lead to inflammation of the liver, causing long‐term liver damage. This damage can progress to liver fibrosis and ultimately to cirrhosis or hepatocellular carcinoma.^[^ ^2^, ^3^ ^]^ While current antiviral treatments effectively suppress HBV replication, they do not cure the infection due to the persistence of covalently closed circular DNA (cccDNA).^[^ ^4^, ^5^, ^6^ ^]^ The cccDNA structure is stable and difficult to eliminate, making it a major hurdle in achieving a cure for chronic HBV. Instead, researchers are focusing on strategies to silence the transcriptional activity of cccDNA, which could be a more feasible approach to control hepatitis B.^[^ ^7^ ^]^

HBV cccDNA exists as a histone‐bound minichromosome, and its transcriptional activity is regulated through various epigenetic mechanisms, including DNA methylation, histone modifications, non‐coding RNA (ncRNA) interference, and chromatin remodeling.^[^ ^7^ ^]^ Among the ncRNAs, long non‐coding RNAs (lncRNAs) are the most abundant and have been found to play a role in regulating gene expression at the transcriptional or post‐transcriptional level.^[^ ^8^, ^9^ ^]^ They can act as signals, decoys, guides, or scaffolds for gene regulation.^[^ ^10^, ^11^ ^]^ Importantly, lncRNAs can act as scaffold to assemble chromatin modification complexes or guide them to specific genomic loci. By doing so, lncRNAs control the deposition of histone marks on chromatin regions, thereby regulating transcriptional activity.^[^ ^10^, ^12^ ^]^ For example, the lncRNA HOTAIR recruits the PRC2 complex to specific target genes genome‐wide, resulting in the trimethylation of H3K27 and the subsequent epigenetic silencing of metastasis suppressor genes.^[^ ^13^, ^14^ ^]^ Similarly, the lncRNA LINC00525 recruits the EZH2 protein to the p21 promoter, leading to an increase in H3K27me3 and the epigenetic inhibition of p21 transcription. This promotes the progression of lung adenocarcinoma.^[^ ^15^ ^]^ Another example is the KSHV‐encoded lncRNA PAN RNA, which acts as a guide RNA to deliver chromatin modification complexes to specific viral genomic locations for viral reactivation.^[^ ^16^ ^]^ Recent studies have identified lncRNAs that cooperate with histone modification enzymes to regulate the transcriptional activity of HBV cccDNA. Examples include LINC01431 and lncDLEU2.^[^ ^17^, ^18^ ^]^ However, the number of lncRNAs known to regulate the transcriptional activity of cccDNA is limited, and the specific mechanisms by which lncRNAs regulate histone modifications are still largely unknown.

Herein, we discovered that viral protein HBV DNA polymerase (HBV DNA pol) upregulates the expression of host lncRNA HOXA‐AS2 after establishing persistent high‐level HBV replication. Subsequently, HOXA‐AS2 directly binds to HBV cccDNA and recruits MTA1‐HDAC1/2 deacetylase complexes, epigenetically suppressing HBV transcription. These findings provide new insights into the epigenetic inhibition of cccDNA transcription by lncRNAs and elucidate a mechanism for self‐limiting HBV replication.

## 2 Results

### 2.1 Screening and Identification of lncRNAs Associated with HBV Replication

**Figure 1:**
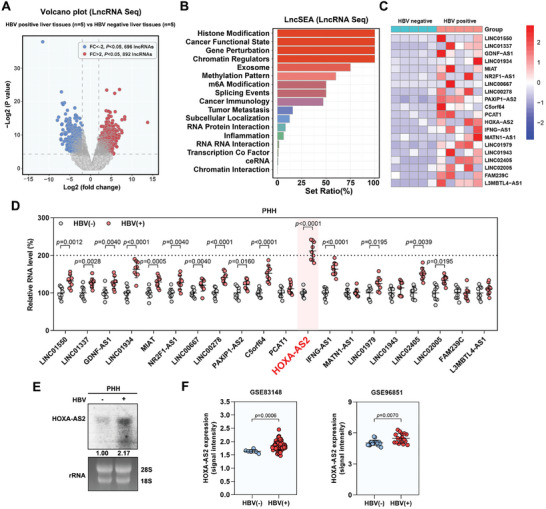
Screening and identification of lncRNAs associated with HBV replication. A) Differential expression of lncRNAs in five pairs of HBV‐positive liver tissues compared to HBV‐negative liver tissues. The volcano plot represents upregulated lncRNAs in red and downregulated lncRNAs in blue. B) Enrichment analysis (LncSEA) of differentially expressed lncRNAs after HBV infection. The top 17 gene sets showing significant enrichment are displayed. C) Heat map representing the expression levels of 20 upregulated lncRNAs associated with histone modification. D) The expression levels of these up‐regulated lncRNAs in uninfected and HBV‐infected PHHs were detected by real‐time PCR. E) PHHs were infected with HBV at 500 vge per cell for 9 days. Subsequently, Northern blot analysis was performed to detect the expression level of HOXA‐AS2 in HBV‐infected cells or uninfected cells. Ribosomal RNAs (28S and 18S) were used as loading controls. F) HBV infection upregulates HOXA‐AS2 expression, data from GSE83148 (control healthy patients, n = 6; HBV‐infected patients, n = 122) and GSE96851 (liver angioma patients, n = 16; HBV‐associated acute liver failure patients, n = 17). For (D), representative data from at least three independent experiments are shown. Data are presented as mean ± SD. Statistical analysis was performed using the Mann–Whitney U test.

To identify lncRNAs that play a crucial role in HBV replication, lncRNA sequencing (lncRNA seq) was performed in five pairs of HBV‐positive liver tissues (HBeAg‐positive chronic hepatitis B (CHB) patients) and HBV‐negative liver tissues (normal liver tissues from patients with biliary related diseases). Through this analysis, a total of 1588 differentially expressed lncRNAs (Log_2_ Fold change > 2; *p* < 0.05; 892 upregulated and 696 downregulated) were identified in HBV‐positive liver tissues (**Figure** **1**A; Figure S1A, Supporting Information). Further analysis using LncSEA enrichment analysis revealed that the differentially expressed lncRNAs were significantly enriched in biological processes related to histone modifications, cancer functional state, gene perturbation, and chromatin regulators (Figure 1B). Considering the importance of histone modification in regulating viral transcription, we focused on the top 20 upregulated lncRNAs in the histone modification gene set for further validation (Figure 1C). Among these 20 lncRNAs, the expression of HOXA‐AS2 was significantly upregulated in HBV‐infected primary human hepatocytes (PHHs) and HepG2‐NTCP cells (Figure 1D; Figure S1B, Supporting Information). This upregulation was further confirmed by northern blot analysis, which showed increased levels of HOXA‐AS2 in HBV‐infected PHHs and HepG2‐NTCP cells (Figure 1E; Figure S1C, Supporting Information). Moreover, analysis of publicly available datasets (GSE83148 and GSE96851) also indicated higher expression of HOXA‐AS2 in HBV‐positive patients compared to HBV‐negative patients (Figure 1F). We simultaneously analyzed the dataset GSE230397, including healthy controls and patients at different stages of HBV infection. In this dataset, HOXA‐AS2 levels were higher in HBV patients compared to healthy controls (*p* = 0.0112). Further analysis of the levels of HOXA‐AS2 at different stages of HBV infection and observed a gradual increase in HOXA‐AS2 levels with the progression of HBV infection (Figure S1D, Supporting Information). These results are consistent with deep sequencing data, suggesting that the increased HOXA‐AS2 may be involved in the regulation of HBV.

### 2.2 HOXA‐AS2 is a Host Restriction Factor that Inhibits cccDNA Transcription and HBV Replication

**Figure 2:**
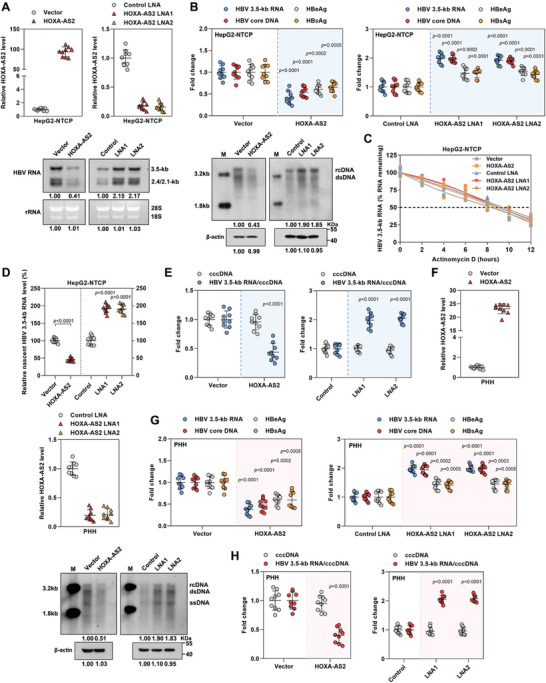
HOXA‐AS2 inhibits cccDNA transcription and HBV replication. A–E) After 24 h of HBV inoculation in HepG2‐NTCP cells, the cells were transduced with lentivirus expressing vector or HOXA‐AS2, or transfected with control locked nucleic acid (LNA) or HOXA‐AS2 LNA for 5 days. (A) Expression of HOXA‐AS2 was measured by real‐time PCR. (B) Levels of intracellular HBV 3.5‐kb RNA and HBV core DNA were analyzed by real‐time PCR. Secreted HBeAg and HBsAg in the culture supernatant were quantified by ELISA. Northern blot and southern blot were performed to assess cellular HBV RNA and DNA, respectively. Ribosomal RNAs (28S and 18S) were used as loading controls. The level of *β*‐actin in the cell lysates (the st1 step of core DNA extraction) was used as a control for cell numbers. The intensity of viral RNA and DNA bands relative to the vector group is indicated below the lanes. (C) HBV‐infected HepG2‐NTCP cells were treated with actinomycin D to inhibit transcription, and the decay of intracellular HBV 3.5‐kb RNA was measured by real‐time PCR at different time points. (D) Cells were treated with EU (5‐ethynyl uridine) (0.2 mmol L^−1^) for 24 h to label newly synthesized RNA, and the newly synthesized EU‐labeled HBV 3.5‐kb RNA was quantified by real‐time PCR. (E) Analysis of HBV cccDNA by real‐time PCR, and the ratios of HBV 3.5‐kb RNA/cccDNA were calculated. F–H) After 24 h of HBV inoculation in Primary human hepatocytes (PHHs), the cells were transduced with lentivirus expressing vector or HOXA‐AS2, or transfected with control locked nucleic acid (LNA) or HOXA‐AS2 LNA for 5 days. (F) Expression of HOXA‐AS2 was measured by real‐time PCR. (G,H) Levels of HBV 3.5‐kb RNA, HBV core DNA, HBeAg, and HBsAg, as well as the HBV 3.5‐kb RNA/cccDNA ratio, were measured. Southern blot was performed to assess cellular HBV DNA, and the level of *β*‐actin in the cell lysates (the st1 step of core DNA extraction) was used as a control for cell numbers. Representative data from at least three independent experiments are shown. Data are presented as mean ± SD. Statistical analysis was performed using the Mann–Whitney U test.

To investigate the role of HOXA‐AS2 in HBV replication, HOXA‐AS2 was overexpressed by lentivirus‐mediated strategy and knocked down by specific locked nucleic acid (LNA) GapmeRs in HBV‐infected HepG2‐NTCP cells. We found that manipulating the expression of HOXA‐AS2 did not have any significant impact on the viability of the cells (**Figure** **2**A; Figure S2A,B, Supporting Information). However, overexpression of HOXA‐AS2 resulted in a significant decrease in the levels of HBV 3.5‐kb RNA, HBV core DNA, HBeAg, and HBsAg, indicating reduced HBV replication. Conversely, knockdown of HOXA‐AS2 led to an increase in these HBV replication markers (Figure 2B). This was further confirmed by northern blot analysis, which showed that overexpression of HOXA‐AS2 reduced the levels of various HBV RNAs, while HOXA‐AS2 knockdown increased their levels (Figure 2B). To determine whether the decreased HBV RNA levels were due to alterations in RNA synthesis or decay, we performed additional experiments. The half‐life of HBV 3.5‐kb RNA was found to be unaffected by HOXA‐AS2, suggesting that HOXA‐AS2 does not influence RNA stability (Figure 2C). However, nascent RNA capture assays revealed that the rate of HBV 3.5‐kb RNA synthesis was decreased in cells overexpressing HOXA‐AS2 and increased in cells with HOXA‐AS2 knockdown (Figure 2D). This indicates that HOXA‐AS2 specifically affects the synthesis of new HBV RNA without impacting its stability. Since the cccDNA is the template for HBV RNA transcription, we also examined the effect of HOXA‐AS2 on the cccDNA level and its transcriptional activity. The ratio of HBV 3.5‐kb RNA to cccDNA was calculated as an indicator of HBV cccDNA transcriptional activity. Interestingly, overexpression of HOXA‐AS2 significantly reduced the transcriptional activity of cccDNA without affecting its overall level (Figure 2E). Similar results were observed in HBV‐infected primary human hepatocytes (Figure 2F‐H). Taken together, the results demonstrate that HOXA‐AS2 functions as a host restriction factor for HBV replication.

Previous studies have indicated that HBV infection leads to the accumulation of oxidative damage.^[^ ^19^, ^20^ ^]^ Further investigation was conducted on the impact of HBV‐induced HOXA‐AS2 on cell fate. Immunofluorescence and flow cytometry analysis revealed that the upregulation of HOXA‐AS2 resulted in a decrease in intracellular reactive oxygen species (ROS) levels induced by HBV (Figure S2C,D, Supporting Information). GSH/GSSG quantification also indicated that overexpression of HOXA‐AS2 enhanced the antioxidant capacity of HBV‐infected cells (Figure S2E, Supporting Information). Additionally, oxidative‐induced DNA damage was assessed by measuring AP sites and double‐strand DNA breaks (DSBs), and it was observed that the overexpression of HOXA‐AS2 significantly reduced HBV‐induced DNA damage (Figure S2F‐I, Supporting Information). These findings suggest that HOXA‐AS2 alleviates HBV‐induced oxidative damage by inhibiting HBV replication.

### 2.3 HOXA‐AS2 Forms an RNA‐DNA Triplex with cccDNA to Inhibit cccDNA Transcription

**Figure 3:**
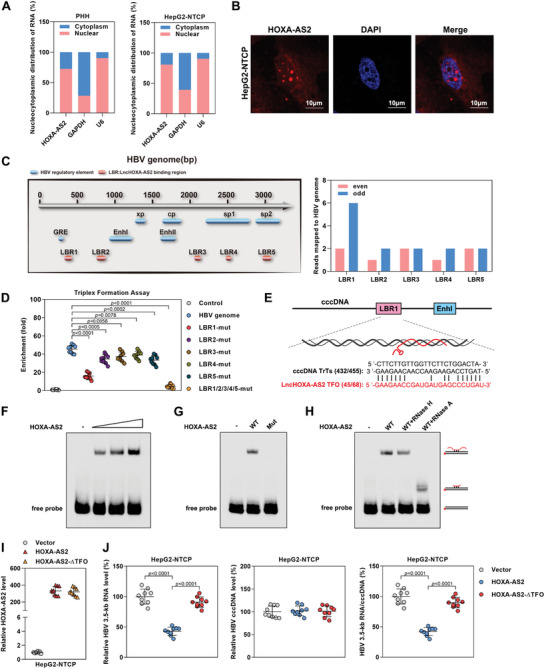
HOXA‐AS2 forms an RNA‐DNA triplex with cccDNA to inhibit cccDNA transcription. A) The levels of HOXA‐AS2 in the cytoplasmic and nuclear fractions of PHHs and HepG2‐NTCP cells were measured by real‐time PCR. Cytoplasmic Glyceraldehyde‐3‐phosphate dehydrogenase (GAPDH) and nuclear snRNA U6 served as markers for fractionation. B) Expression of HOXA‐AS2 in HepG2‐NTCP cells was detected by RNA‐FISH. Red represents HOXA‐AS2, and blue represents DAPI staining. Scale bar = 10 µm. FISH stands for fluorescence in situ hybridization. C) Left: Mapping of the lncHOXA‐AS2 binding cccDNA region (LBR) onto the HBV genome. Right: HepG2‐NTCP cells were infected with HBV for 6 days, ChIRP‐sequencing was performed to analyze HOXA‐AS2‐associated cccDNA. Even and odd probe sets were used for HOXA‐AS2 ChIRP. D) Biotinylated HOXA‐AS2 was incubated with HBV genome or its deletion mutant (LBR1/2/3/4/5‐mut). The HOXA‐AS2 associated with HBV was measured by real‐time PCR after binding to streptavidin beads. E) Triplex‐forming oligonucleotides (TFO) binding sites within HOXA‐AS2 and triplex target sites (TrTs) within cccDNA are indicated. F) HOXA‐AS2 forms DNA‐RNA triplexes with LBR1. Increasing amounts (40‐, 80‐ or 160‐fold molar excess) of HOXA‐AS2 (1/139) were incubated with double‐stranded oligonucleotide comprising LBR1 fragment (425/458) of HBV genome, and formation of RNA‐DNA triplexes was monitored by EMSA. G) Sequence‐specific interaction of HOXA‐AS2 with LBR1. EMSA shows the mobility of biotinylated LBR1 fragment (425/458) of HBV genome after incubation with wild‐type or mutant HOXA‐AS2 (1/139). H) Reactions containing labeled LBR1 fragment of HBV genome and an 80‐molar excess of HOXA‐AS2 (1/139) were treated with 0.5 U of RNase H or with 0.5 ng RNase A for 30 min at room temperature. I,J) HBV‐infected HepG2‐NTCP cells were transfected with the HOXA‐AS2 plasmid or its TFO mutant for 5 days. (I) Expression of HOXA‐AS2 was measured by real‐time PCR. (J) The levels of HBV 3.5‐kb RNA, cccDNA, and the HBV 3.5‐kb RNA/cccDNA ratio were measured. For (A), (D), (I) and (J), representative data from at least three independent experiments are shown. Data are presented as mean ± SD. Statistical analysis was performed using the Mann–Whitney U test.

HOXA‐AS2 is a lncRNA that is located on chromosome 7p15.2 and has a full‐length transcript of 1048 nt (Figure S3A, Supporting Information). We aimed to determine the subcellular localization of HOXA‐AS2 to gain insight into its regulatory mechanism. Through cellular fractionation and RNA fluorescence in situ hybridization (RNA‐FISH) assays, it was found that HOXA‐AS2 is predominantly localized in the nucleus (**Figure** **3**A,B). Nuclear‐localized lncRNAs can form specialized structures, such as RNA‐DNA heteroduplexes and RNA‐DNA triplexes, which play a role in recruiting chromatin‐modifying enzymes to specific genomic regions.^[^ ^21^, ^22^, ^23^ ^]^ To investigate the interaction between HOXA‐AS2 and the cccDNA of HBV, cccDNA chromatin isolation by RNA purification (ChIRP) assays was performed in HBV‐infected HepG2‐NTCP cells. The results revealed that HOXA‐AS2 binds to five regions within the cccDNA, designated as LBR1‐5 (lncHOXA‐AS2 binding regions) (Figure 3C; Figure S3B and Table S1, Supporting Information). To determine the specific region of cccDNA critical for its interaction with HOXA‐AS2, HBV genome mutants with deletions in the LBR1‐5 regions (referred to as LBR1/2/3/4/5‐mut) were constructed. The results showed that the interaction between HOXA‐AS2 and HBV was most significantly impaired when the LBR1 region of HBV genome was deleted (Figure 3D). This suggests that the LBR1 region of cccDNA may be primarily responsible for binding to HOXA‐AS2.

Previous research has indicated that triplex‐forming oligonucleotide sites (TFOs) are often enriched with sequences containing GA repeats. These GA‐rich sequences in target genes and lncRNAs can form triplex structures through the formation of Hoogsteen bonds between RNA and DNA.^[^ ^21^, ^22^, ^23^ ^]^ Interestingly, through sequence alignment analysis, potential TFO within HOXA‐AS2 and corresponding triplex targeting sites (TrTs) in the LBR1 region of cccDNA were identified (Figure 3E). To examine the potential of HOXA‐AS2 to form RNA‐DNA triplexes, an in vitro triplex pull‐down assay was conducted using biotin‐labeled HOXA‐AS2 WT (HOXA‐AS2 (1/139)) or HOXA‐AS2 Mut (deletion of the TFO region (45/68) in HOXA‐AS2 WT). The results revealed that HOXA‐AS2 WT was able to bind to the LBR1 fragment (425/458) of HBV genome, whereas the HOXA‐AS2 Mut could not (Figure S3C, Supporting Information). As an alternative approach, we monitored triplex formation by electrophoretic mobility shift assays (EMSA). In accord with the pull‐down data, HOXA‐AS2 forms a low‐mobility RNA‐DNA complex with biotinylated LBR1 fragment of HBV genome in a concentration‐dependent manner, indicating an interaction between HOXA‐AS2 and LBR1 (Figure 3F). Furthermore, it was found that the deletion of the TFO region in HOXA‐AS2 (HOXA‐AS2 Mut) prevented its formation of a low‐mobility RNA‐DNA complex with biotinylated LBR1 fragment, suggesting that the TFO region of HOXA‐AS2 plays a crucial role in its interaction with LBR1 (Figure 3G). Moreover, upon treatment with RNase A, the mobility of the HOXA‐AS2/LBR1 complex was increased, indicating that the part of RNA that contacts DNA was shielded from degradation. Treatment with RNase H did not affect the mobility of the complex, excluding the possibility that HOXA‐AS2 interacts with LBR1 by forming an RNA‐DNA heteroduplexes (Figure 3H). Together, these results suggest that HOXA‐AS2 can form a triplex with LBR1 region of cccDNA.

Subsequently, HBV‐infected HepG2‐NTCP cells were transfected with HOXA‐AS2 and a mutant form of HOXA‐AS2 with a TFO deletion (HOXA‐AS2‐ΔTFO). The deletion of the TFO sequence significantly attenuated the ability of HOXA‐AS2 to repress HBV transcription and replication (Figure 3I,J; Figure S3D, Supporting Information). In conclusion, these findings provide evidence that HOXA‐AS2 forms a triplex with cccDNA through the TFO site and inhibits cccDNA transcription.

### 2.4 MTA1 Mediates HOXA‐AS2 Binding to the MTA1‐HDAC1/2 Complex

**Figure 4:**
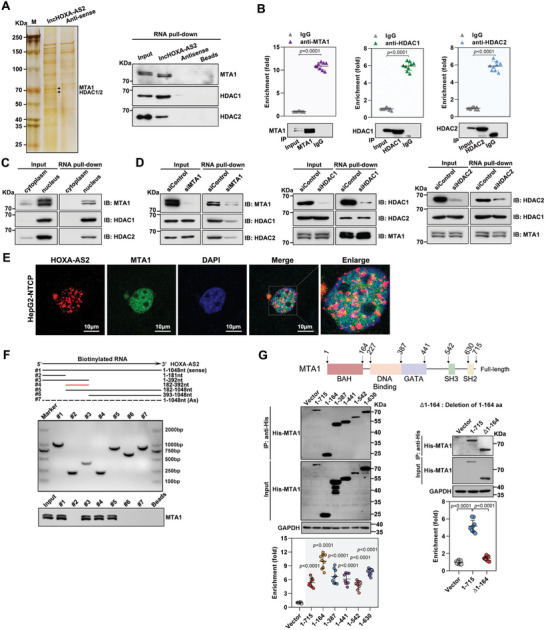
MTA1 mediates HOXA‐AS2 binding to the MTA1‐HDAC1/2 complex. A) Left: Streptavidin RNA pull‐down assay and silver staining were used to detect the specific associations of proteins with biotinylated‐HOXA‐AS2. The arrow indicated the HOXA‐AS2‐specific band compared with antisense RNA. Right: RNA pull‐down assay and western blot were performed to detect the specific associations of HOXA‐AS2 with MTA1/HDAC1/HDAC2. B) RNA immunoprecipitation (RIP) assays were conducted to determine the association of MTA1/HDAC1/HDAC2 with endogenous HOXA‐AS2 in HepG2‐NTCP cells. Normalized data are presented as relative fold enrichment compared to the control group. C) RNA‐pull down assay was used to detect the interaction of biotin‐labeled HOXA‐AS2 with MTA1‐HDAC1/2 complex in the cytoplasm or nucleus of HepG2‐NTCP cells. D) RNA pull‐down assay was performed to assess the capacity of HOXA‐AS2 to bind to the MTA1‐HDAC1/2 complex after silencing MTA1/HDAC1/HDAC2. E) Immunofluorescence staining was used to visualize HOXA‐AS2 and MTA1 in HepG2‐NTCP cells. Red represents HOXA‐AS2, green represents MTA1, and blue represents DAPI staining. Scale bar = 10 µm. F) Schematic of truncated HOXA‐AS2 (upper), with full‐length HOXA‐AS2 and truncated versions confirmed by PCR (middle). RNA pull‐down assay was conducted to detect the binding of MTA1 protein with truncated‐HOXA‐AS2 (bottom). G) RIP assay for the in vivo interaction between HOXA‐AS2 and MTA1. HepG2‐NTCP cells were transfected with HOXA‐AS2 and plasmids expressing different His‐tagged‐MTA1 truncates. RIP assay was performed using anti‐His antibody at day 3 post transfection. Normalized data are presented as relative fold enrichment compared to the control group. For (B) and (G), representative data from at least three independent experiments are shown. Data are presented as mean ± SD. Statistical analysis was performed using the Mann–Whitney U test.

LncRNAs are considered to exert their functions through RNA‐interacting proteins that regulate gene expression by various mechanisms.^[^ ^24^ ^]^ To investigate the proteins that interact with HOXA‐AS2 and mediate its function, an RNA pull‐down assay was performed using biotin‐labeled HOXA‐AS2. Through mass spectrometry and bioinformatics analysis, a total of 39 nuclear proteins were identified as potential HOXA‐AS2‐associated proteins (Figure S4A, Supporting Information). Nuclear‐localized lncRNAs have been reported to act as a prominent layer of transcriptional regulation, often by collaborating with chromatin modification complexes.^[^ ^11^, ^25^, ^26^ ^]^ Interestingly, functional enrichment analysis revealed that histone deacetylation was significantly enriched among these proteins. Among the biological process of histone deacetylation, metastasis associated 1 (MTA1), histone deacetylase 1 (HDAC1), and histone deacetylase 1 (HDAC2) were the three identified proteins with the highest binding abundance (Figure S4A, Supporting Information). Furthermore, it was observed that the overexpression or silencing of HOXA‐AS2 did not affect the expression levels of MTA1, HDAC1, and HDAC2 (Figure S4B, Supporting Information). Subsequently, the interaction between HOXA‐AS2 and MTA1, HDAC1, and HDAC2 was confirmed through RNA pull‐down and RIP assays (**Figure** **4**A,B). Additionally, fractionation of cytoplasmic and nuclear fractions of HepG2‐NTCP cells demonstrated that the interaction between HOXA‐AS2 and MTA1/HDAC1/HDAC2 predominantly occurs in the nucleus (Figure 4C; Figure S4C, Supporting Information). MTA1, HDAC1, and HDAC2 are known to form deacetylase complexes with histone deacetylase activity and transcriptional inhibition activity.^[^ ^27^ ^]^ Co‐immunoprecipitation and immunofluorescence assays confirmed the interaction and co‐localization of MTA1, HDAC1, and HDAC2 (Figure S4D,E, Supporting Information). To determine which subunit of the MTA1‐HDAC1/2 deacetylase complex is responsible for mediating the binding to HOXA‐AS2, individual silencing of MTA1, HDAC1, and HDAC2 was performed in HepG2‐NTCP cells. RNA pull‐down experiments were conducted to assess the ability of HOXA‐AS2 to bind the MTA1‐HDAC1/2 complex. Interestingly, the silencing of MTA1 significantly reduced the binding of HOXA‐AS2 to the MTA1‐HDAC1/2 complex, while the silencing of HDAC1 or HDAC2 did not have the same effect (Figure 4D). Furthermore, immunofluorescence assays were performed to confirm the co‐localization of HOXA‐AS2 and MTA1 in HepG2‐NTCP cells (Figure 4E), providing additional evidence that MTA1 mediates the binding of HOXA‐AS2 to the MTA1‐HDAC1/2 complex.

To determine the specific region of HOXA‐AS2 that interacts with MTA1, the secondary structure of HOXA‐AS2 was predicted using an online RNA structure prediction software (http://www.unafold.org/). Based on the predicted secondary structure, truncated fragments of HOXA‐AS2 were constructed (Figure S4F, Supporting Information). RNA pull‐down assays were performed using these truncated segments, and it was found that the region spanning nucleotides 182‐392 of HOXA‐AS2, which forms a flat stem‐loop structure, mediates the interaction with MTA1 (Figure 4F). Simultaneously, efforts were made to identify the key regions in MTA1 responsible for the interaction with HOXA‐AS2. Previous studies suggested that the BAH domain located at the N‐terminal portion of MTA1 may serve as the primary RNA‐binding motif of MTA1.^[^ ^28^ ^]^ Further truncation analysis confirmed that the BAH domain, comprising amino acids 1‐164 of MTA1, was primarily responsible for the HOXA‐AS2‐MTA1 interaction. Additionally, a mutant form of MTA1 with a deletion of amino acids 1‐164 (MTA1 (Δ1‐164)) lost its ability to interact with HOXA‐AS2 (Figure 4G; Figure S4G, Supporting Information). Overall, these findings indicate that the interaction between the flat stem‐loop structure of HOXA‐AS2 and the BAH domain of MTA1 is crucial for the formation of the HOXA‐AS2‐MTA1‐HDAC1/2 complex.

### 2.5 MTA1‐HDAC1/2 Deacetylase Complex Mediates the Inhibitory Effect of HOXA‐AS2 on cccDNA Transcription

**Figure 5:**
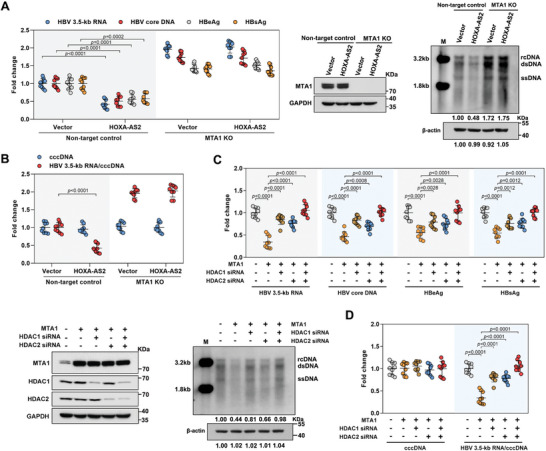
MTA1‐HDAC1/2 deacetylase complex mediates the inhibitory effect of HOXA‐AS2 on cccDNA transcription. A,B) HepG2‐NTCP cells with MTA1 knockout were infected with HBV for 24 h and then transduced with lentivirus expressing vector or HOXA‐AS2 for 5 days. (A) Levels of intracellular HBV 3.5‐kb RNA and HBV core DNA were analyzed by real‐time PCR. Secreted HBeAg and HBsAg in the culture supernatant were quantified by ELISA (left). MTA1‐knocked out sub‐clones in HepG2‐NTCP cells were constructed and the knockout efficiency of MTA1 was validated by western blot. GAPDH were used as loading control (middle). Southern blot was performed to assess cellular HBV DNA, and the level of *β*‐actin in the cell lysates (the st1 step of core DNA extraction) was used as a control for cell numbers (right). (B) Analysis of HBV cccDNA by real‐time PCR, and the ratios of HBV 3.5‐kb RNA/cccDNA were calculated. C,D) HepG2‐NTCP cells were infected with HBV for 24 h and then transfected with MTA1 expression plasmids and HDAC1/2 siRNA for 5 days. (C) Levels of intracellular HBV 3.5‐kb RNA and HBV core DNA were analyzed by real‐time PCR. Secreted HBeAg and HBsAg in the culture supernatant were quantified by ELISA. The expression levels of MTA1, HDAC1, and HDAC2 were detected using western blot. Southern blot was performed to assess cellular HBV DNA, and the level of *β*‐actin in the cell lysates (the st1 step of core DNA extraction) was used as a control for cell numbers. (D) Analysis of HBV cccDNA by real‐time PCR, and the ratios of HBV 3.5‐kb RNA/cccDNA were calculated.

The MTA1‐HDAC1/2 deacetylase complex is a histone modifier and inhibits gene expression.^[^ ^27^ ^]^ To investigate whether the MTA1‐HDAC1/2 deacetylase complex is involved in the inhibitory effect of HOXA‐AS2 on cccDNA transcription, MTA1‐knockout sub‐clones were generated in HepG2‐NTCP cells using the CRISPR/Cas9 system. The successful knockout of MTA1 was confirmed by western blot analysis (**Figure** **5**A). The inhibition of HBV replication and cccDNA transcriptional activity induced by overexpression of HOXA‐AS2 was found to be significantly blocked in MTA1 knockout cells (Figure 5A,B; Figure S5A, Supporting Information). This suggests that MTA1 is essential for the inhibitory effect of HOXA‐AS2 on cccDNA transcription. Furthermore, overexpression of MTA1 alone resulted in significant inhibition of HBV replication and cccDNA transcriptional activity. Conversely, knockdown of HDAC1 or HDAC2 attenuated the suppressive effect of MTA1 on HBV replication (Figure 5C,D). Moreover, simultaneous knockdown of both HDAC1 and HDAC2 further alleviated the MTA1‐induced inhibition of HBV (Figure 5C,D). These findings collectively indicate that the MTA1‐HDAC1/2 deacetylase complex is involved in mediating the inhibitory effect of HOXA‐AS2 on cccDNA transcription.

### 2.6 HOXA‐AS2 Increases the Occupancy of the MTA1‐HDAC1/2 Complex on HBV cccDNA to Inhibit cccDNA Transcription

**Figure 6:**
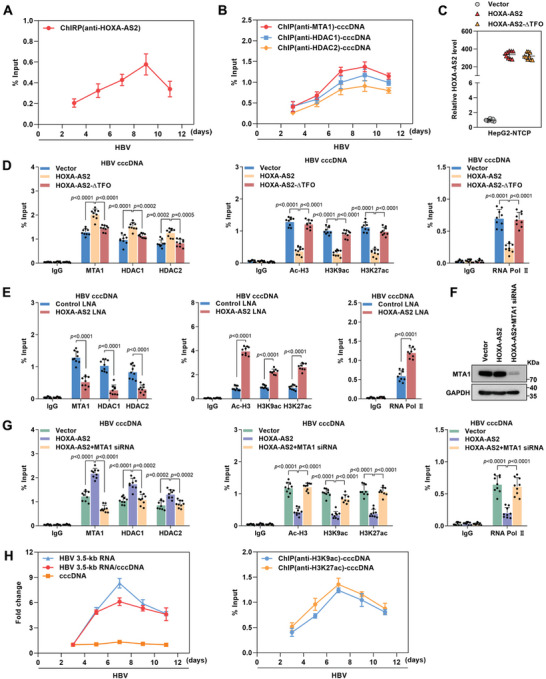
HOXA‐AS2 increases the occupancy of the MTA1‐HDAC1/2 complex on HBV cccDNA to inhibit cccDNA transcription. A,B) HBV‐infected HepG2‐NTCP cells were harvested at the indicated time points. Cells were analyzed for HOXA‐AS2‐associated cccDNA using ChIRP assay (A) and the occupancy of the MTA1‐HDAC1/2 complex on HBV cccDNA using ChIP assay (B). C–E) HBV‐infected HepG2‐NTCP cells were transfected with HOXA‐AS2 or HOXA‐AS2‐ΔTFO expression plasmid or treated with HOXA‐AS2 LNA for 5 days. (C) Expression of HOXA‐AS2 was measured by real‐time PCR. D,E) The enrichment of the MTA1‐HDAC1/2 complex, H3K9ac, H3K27ac, and RNA polymerase II on cccDNA was detected using ChIP assays. F,G) HBV‐infected HepG2‐NTCP cells were transfected with HOXA‐AS2 plasmid and MTA1 siRNA for 5 days. (F) The expression level of MTA1 was detected using western blot. (G) The enrichment of the MTA1‐HDAC1/2 complex, H3K9ac, H3K27ac, and RNA polymerase II on cccDNA was detected using ChIP assays. H) HBV‐infected HepG2‐NTCP cells were analyzed for the levels of HBV 3.5‐kb RNA, cccDNA, HBV 3.5‐kb RNA/cccDNA, and the occupancy of H3K9ac and H3K27ac on cccDNA at different time points. ChIP results are expressed as % of input. Representative data from at least three independent experiments are shown. Data are presented as mean ± SD. Statistical analysis was performed using the Mann–Whitney U test.

The MTA1‐HDAC1/2 deacetylase complex catalyzes the deacetylation of H3K9 and H3K27 and represses gene transcription.^[^ ^27^, ^29^, ^30^ ^]^ To investigate the impact of HOXA‐AS2 on the occupancy of the MTA1‐HDAC1/2 deacetylase complex on cccDNA, ChIRP and Chromatin immunoprecipitation (ChIP) assays were performed. These assays revealed that the binding pattern of HOXA‐AS2 to cccDNA was consistent with that of the MTA1‐HDAC1/2 deacetylase complex (**Figure** **6**A,B). Further analysis was carried out to understand the role of HOXA‐AS2 in the binding of the MTA1‐HDAC1/2 deacetylase complex to cccDNA. HBV‐infected HepG2‐NTCP cells were transfected with either the wild‐type HOXA‐AS2 plasmid or the HOXA‐AS2‐∆TFO plasmid, which lacks the ability to bind to cccDNA (Figure 6C; Figure S6A, Supporting Information). It was observed that the wild‐type HOXA‐AS2 increased the recruitment of the MTA1‐HDAC1/2 deacetylase complex to cccDNA, leading to decreased levels of H3K9ac, H3K27ac, and RNA polymerase II on cccDNA. However, the HOXA‐AS2‐∆TFO mutant failed to induce the aforementioned effects (Figure 6D; Figure S7A, Supporting Information). Conversely, silencing of HOXA‐AS2 resulted in decreased recruitment of the MTA1‐HDAC1/2 deacetylase complex to cccDNA, accompanied by increased levels of acetylation and RNA polymerase II on cccDNA (Figure 6E; Figure S7B, Supporting Information). Notably, the knockdown of MTA1 significantly rescued the HOXA‐AS2‐initiated enrichment of MTA1‐HDAC1/2 deacetylase complex and increased levels of acetylation and RNA polymerase II on cccDNA (Figure 6F,G; Figure S7C, Supporting Information). Furthermore, ChIP assay results demonstrated that the binding of HOXA‐AS2 and the MTA1‐HDAC1/2 deacetylase complex to cccDNA increased at the fastest rate on day 7 after HBV infection when the levels of H3K9ac, H3K27ac, and cccDNA transcriptional activity were observed to decrease. This suggests that the recruitment of HOXA‐AS2 and the MTA1‐HDAC1/2 deacetylase complex to cccDNA might contribute to preventing overactivation of cccDNA transcription (Figure 6A,B,H). Overall, these findings indicate that HOXA‐AS2 recruits the MTA1‐HDAC1/2 deacetylase complex to cccDNA, leading to the inhibition of cccDNA transcription.

### 2.7 HOXA‐AS2 Inhibits cccDNA Transcription In Vivo

**Figure 7:**
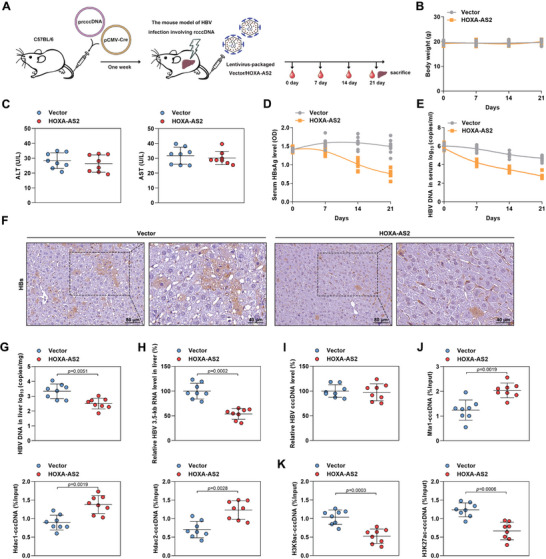
HOXA‐AS2 inhibits cccDNA transcription in vivo. A) Schematic of experimental plans. C57BL/6 mice were injected with prcccDNA and pCMV‐Cre. After one week, the mice were injected with a lentivirus‐packaged vector or HOXA‐AS2 for 21 days. Blood samples were collected at 0, 7, 14, and 21 days, and liver tissues were collected at day 21 for analysis. B) Monitoring of body weight in mice every 7 days. C) Measurement of alanine aminotransferase (ALT) and aspartate aminotransferase (AST) levels in serum on day 21 using a colorimetric microplate assay. D) Detection of HBsAg levels in serum by ELISA. E) Measurement of serum HBV genomic DNA levels by real‐time PCR. F) Analysis of HBs expression in liver tissues by immunohistochemistry. Scale bar = 80 µm (left panel) or 40 µm (right panel). G–I) Determination of HBV DNA, HBV 3.5‐kb RNA, and cccDNA levels in liver tissues by real‐time PCR. J,K) Analysis of the levels of Mta1, Hdac1, Hdac2, H3K9ac, and H3K27ac associated with cccDNA using ChIP assay. For (B–E) and (G–K), the data were presented as mean ± SD, n = 8 in each group. Statistical analyses were performed using Student's t‐test.

To confirm the inhibitory effect of HOXA‐AS2 on cccDNA transcription in vivo, a mouse model of HBV infection was utilized.^[^ ^31^ ^]^ The mice were hydrodynamically co‐injected with precursor recombinant cccDNA (prcccDNA) and pCMV‐Cre, resulting in the accumulation of nuclear cccDNA organized as a minichromosome in the mouse liver. Subsequently, mice were injected with lentivirus packaged with either control vector or HOXA‐AS2 for 21 days to assess the impact of HOXA‐AS2 overexpression on HBV (**Figure** **7**A), We monitored body weight, measured alanine aminotransferase (ALT) and aspartate aminotransferase (AST) levels to assess liver function, and conducted FISH to detect the expression efficiency of HOXA‐AS2. As shown in Figure S8A (Supporting Information), lentivirus resulted in elevated HOXA‐AS2 expression in mouse liver, and HOXA‐AS2 treatment did not cause significant hepatotoxicity (Figure 7B,C). Consistent with the results observed in HBV‐infected HepG2‐NTCP cells, overexpression of HOXA‐AS2 in the mouse model significantly reduced the levels of HBsAg and HBV DNA in the serum (Figure 7D,E), as well as the levels of HBs, HBc and HBV DNA in the liver (Figure 7F,G; Figure S8A, Supporting Information). Furthermore, overexpression of HOXA‐AS2 led to a significant decrease in intrahepatic HBV 3.5‐kb RNA levels, without affecting cccDNA levels (Figure 7H,I). These results indicate that HOXA‐AS2 can exert an inhibitory effect on cccDNA transcription in vivo.

To investigate whether the Mta1‐Hdac1/2 deacetylase complex is involved in the inhibitory effect of HOXA‐AS2 on cccDNA transcription in HBV infection mouse model, we conducted a comparison of the human MTA1 and mouse Mta1 sequences using the UniProt database (ID: Q13330 and Q8K4B0), The results revealed a high similarity of 97.1% in their amino acid sequences. Furthermore, we used AlphaFold2 to perform structure prediction and alignment for both human MTA1 and mouse Mta1. The results showed a RMSD (Root Mean Square Deviation) value of 3.281 Å between the two proteins, indicating significant structural similarity (Figure S8B, Supporting Information). Finally, we performed experimental validation using mouse‐derived Hepa 1–6 cells, and the RNA pull‐down results confirmed the capability of human HOXA‐AS2 to bind to mouse Mta1 (Figure S8C, Supporting Information). We then conducted cccDNA‐ChIP assay in liver tissue, which revealed that overexpression of HOXA‐AS2 increased the binding of the Mta1‐Hdac1/2 deacetylase complex to cccDNA. This was accompanied by a decrease in H3K9ac and H3K27ac levels on cccDNA (Figure 7J,K). Taken together, these findings confirm that HOXA‐AS2, as a host restriction factor, limits cccDNA transcription in hepatocytes by recruiting the Mta1‐Hdac1/2 deacetylase complex in vivo.

### 2.8 HBV DNA pol Induces HOXA‐AS2 Expression via AP‐2*α*

**Figure 8:**
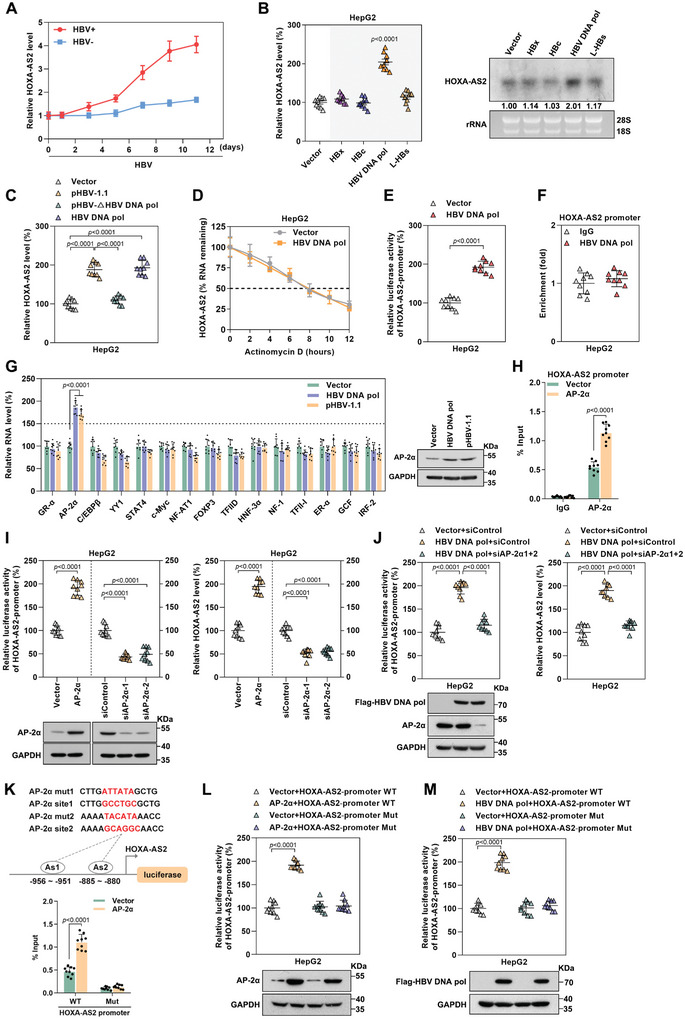
HBV DNA pol induces HOXA‐AS2 expression via AP‐2*α*. A) The level of HOXA‐AS2 was detected by real‐time PCR in HBV‐infected HepG2‐NTCP cells or uninfected cells at different time points. B) HepG2 cells were transfected with different HBV protein expression plasmids for 3 days, and the level of HOXA‐AS2 was measured using real‐time PCR and northern blot. Ribosomal RNAs (28S and 18S) served as loading controls. C) HepG2 cells were transfected with HBV‐1.1 plasmid (pCH9/3091) or its HBV DNA pol mutant or HBV DNA pol expression plasmid for 3 days, and the level of HOXA‐AS2 was detected using real‐time PCR. D) HepG2 cells were transfected with the Flag‐HBV DNA pol expression plasmid for 3 days and treated with actinomycin D for different durations. The stability of HOXA‐AS2 was detected by real‐time PCR. E) HepG2 cells were transfected with the HOXA‐AS2 promoter and Flag‐HBV DNA pol expression plasmid, and the activity of the HOXA‐AS2 promoter was measured using a dual luciferase assay on day 2 post‐transfection. F) HepG2 cells were transfected with the HOXA‐AS2 promoter and Flag‐HBV DNA pol expression plasmid, and the enrichment of HBV DNA pol on the HOXA‐AS2 promoter was determined by ChIP assay. G) HepG2 cells were transfected with vector, Flag‐HBV DNA pol, and HBV‐1.1 plasmid (pCH9/3091) for 3 days, respectively, and the levels of transcription factors were measured using real‐time PCR. The level of AP‐2*α* was determined by western blot. H) HepG2 cells were co‐transfected with the AP‐2*α* expression plasmid and the HOXA‐AS2 promoter plasmid. The association of AP‐2*α* with the HOXA‐AS2 promoter was examined by ChIP assay. I) HepG2 cells were transfected with AP‐2*α* expression plasmid or AP‐2*α* siRNAs, and the HOXA‐AS2 promoter activity was measured using a dual luciferase assay (left). The RNA level of HOXA‐AS2 was detected using real‐time PCR (right), and the expression level of AP‐2*α* was detected using western blot (bottom). J) HepG2 cells were transfected with Flag‐HBV DNA pol and AP‐2*α* siRNAs, and the activity of the HOXA‐AS2 promoter was measured using a dual luciferase assay (left). The RNA level of HOXA‐AS2 was detected using real‐time PCR (right), and the level of Flag‐HBV DNA pol and AP‐2*α* was detected by western blot (bottom). K) Diagram illustrating the binding sites of AP‐2*α* in the HOXA‐AS2 promoter (upper). HepG2 cells were co‐transfected with the AP‐2*α* expression plasmid and the HOXA‐AS2 promoter wild‐type (WT) plasmid or its AP‐2*α* binding site mutant (Mut) plasmid. The association of AP‐2*α* with the HOXA‐AS2 promoter was examined by ChIP assay (bottom). L) HepG2 cells were co‐transfected with the AP‐2*α* expression plasmid and the HOXA‐AS2 promoter wild‐type (WT) plasmid or its AP‐2*α* binding site mutant (Mut) plasmid. The HOXA‐AS2 promoter activity was measured using a dual luciferase assay (upper), and the level of AP‐2*α* was detected by western blot (bottom). M) HepG2 cells were transfected with the Flag‐HBV DNA pol expression plasmid and the HOXA‐AS2 promoter WT plasmid or HOXA‐AS2 promoter Mut plasmid. The HOXA‐AS2 promoter activity was measured using a dual luciferase assay (upper), and the level of Flag‐HBV DNA pol was detected by western blot (bottom). Representative data from at least three independent experiments are shown. Data are presented as mean ± SD. Statistical analysis was performed using the Mann–Whitney U test.

To investigate how HBV induces the expression of HOXA‐AS2, the dynamic changes of HOXA‐AS2 levels during natural HBV infection were examined. Interestingly, we found that HOXA‐AS2 expression was not induced immediately after HBV infection, but was upregulated ≈7 days after HBV infection, suggesting HOXA‐AS2 might play an important role to counteract viral replication when establishment of persistent high level HBV replication (**Figure** **8**A). Previous studies have reported that viral proteins can regulate the expression of various host factors.^[^ ^18^, ^32^, ^33^ ^]^ Through real‐time PCR and northern blot analysis, it was found that HBV DNA pol significantly increased the RNA levels of HOXA‐AS2, while other viral proteins had no significant effect (Figure 8B; Figure S9A, Supporting Information). Subsequent to the deletion of HBV DNA pol (start codon mutation causing HBV DNA pol deletion, HBV‐∆HBV DNA pol), the upregulation of HOXA‐AS2 induced by HBV infection was attenuated, indicating the crucial role of HBV DNA pol in upregulating HOXA‐AS2 expression (Figure 8C; Figure S9B, Supporting Information). In addition, the half‐life of HOXA‐AS2 RNA was found to be unaffected by overexpression or deletion of HBV DNA pol (Figure 8D; Figure S9C,D, Supporting Information). Luciferase reporter assays further showed that HBV DNA pol increased the activity of the HOXA‐AS2 promoter (Figure 8E; Figure S9E, Supporting Information). However, ChIP assays revealed that HBV DNA pol did not directly bind to the HOXA‐AS2 promoter (Figure 8F; Figure S9F, Supporting Information). This implies that HBV DNA pol may indirectly regulate the expression of HOXA‐AS2. Bioinformatics analysis using PROMO predicted that several transcription factors could potentially bind to the HOXA‐AS2 promoter region. Among them, the transcription factor AP‐2*α* was found to be significantly increased at the mRNA and protein levels in cells expressing HBV DNA pol or HBV (Figure 8G). Consistently, compared to wild‐type HBV virus, HBV DNA pol‐deficient virus no longer effectively promotes the expression of AP‐2*α* (Figure S9G, Supporting Information). Further investigation revealed that HBV DNA pol can enhance the stability of AP‐2*α* mRNA, and this phenomenon is abolished upon HBV DNA pol deficiency (Figure S9H,I, Supporting Information). Additional RIP assays confirmed the interaction between HBV DNA pol and AP‐2*α* mRNA (Figure S9J, Supporting Information). These findings suggest that HBV DNA pol binds to and stabilizes AP‐2*α* mRNA, promoting its translation.

AP‐2*α* is a transcription factor that belongs to the activator protein 2 (TFAP2) family, and it regulates gene expression by binding to the promoter of target genes containing AP‐2 binding sites (GCC(N)3/4GGC).^[^ ^34^, ^35^ ^]^ To further investigate the role of AP‐2*α* in HOXA‐AS2 transcription, a ChIP assay was performed and it was found that AP‐2*α* could bind to the HOXA‐AS2 promoter (Figure 8H; Figure S9K, Supporting Information). Consistent with this finding, overexpression of AP‐2*α* significantly increased the promoter activity and RNA level of HOXA‐AS2, as demonstrated by luciferase reporter assays and real‐time PCR. Conversely, knockdown of AP‐2*α* had the opposite effect, resulting in decreased HOXA‐AS2 promoter activity and RNA levels (Figure 8I). These results indicate that AP‐2*α* promotes the transcriptional activity of HOXA‐AS2. Moreover, knockdown of AP‐2*α* significantly attenuated the promotion effect of HBV DNA pol on HOXA‐AS2 promoter activity and RNA levels (Figure 8J). Bioinformatics analysis using PROMO identified two potential AP‐2*α* binding sites in the HOXA‐AS2 promoter. Mutation of the two AP‐2*α* binding sites on the HOXA‐AS2 promoter (HOXA‐AS2‐promoter Mut) significantly disrupted the binding between AP‐2*α* and the HOXA‐AS2 promoter (Figure 8K), resulting in a significant reduction in the enhancing effect of AP‐2*α* on the activity of the HOXA‐AS2 promoter (Figure 8L). These results suggest that AP‐2*α* acts as a transcription factor by directly binding to the promoter of HOXA‐AS2 to promote its transcription. Finally, HBV DNA pol no longer upregulated the activity of the mutant HOXA‐AS2 promoter, indicating that HBV DNA pol promotes the expression of HOXA‐AS2 in an AP‐2*α*‐dependent manner (Figure 8M). In summary, these findings suggest that HBV DNA pol transcriptionally promotes HOXA‐AS2 expression by increasing the expression of AP‐2*α* and facilitating its binding to the HOXA‐AS2 promoter.

## 3 Discussion

Accumulating evidence suggests that epigenetic mechanisms, such as DNA methylation, histone modification, non‐coding RNA (ncRNA) interference, and chromatin remodeling, greatly influence the transcriptional activity of covalently closed circular DNA (cccDNA) in hepatitis B virus (HBV) infections.^[^ ^7^ ^]^ In particular, long non‐coding RNAs (lncRNAs) have been identified as the most abundant type of ncRNAs,^[^ ^8^, ^9^ ^]^ and their role in regulating gene transcription through recruitment of chromatin modification complexes has been well‐established.^[^ ^10^, ^12^ ^]^ However, the specific lncRNAs involved in regulating the transcriptional activity of cccDNA in HBV infections are still limited. Therefore, our study aimed to identify and characterize differentially expressed lncRNAs following HBV infection through lncRNA sequencing. Through functional enrichment analysis, we found that the differentially expressed lncRNAs were significantly enriched in histone modification‐related biological processes. As histone modification on cccDNA has been shown to regulate viral transcription,^[^ ^36^ ^]^ we further selected the top 20 up‐regulated lncRNAs in the histone modification gene set for validation in vitro. Among these lncRNAs, we observed a significant upregulation of HOXA cluster antisense RNA 2 (HOXA‐AS2) following HBV infection. This finding suggests that HOXA‐AS2 may play a role in the replication process of HBV.

We conducted experiments using the HepG2‐NTCP and PHH models of HBV natural infection to investigate the role of HOXA‐AS2 in HBV replication. Our results consistently demonstrated that HOXA‐AS2 is an important host restriction lncRNA that is upregulated by HBV and has the ability to inhibit cccDNA transcription. Previous studies have suggested that lncRNAs can exert their functions by forming RNA‐DNA triplexes or RNA‐DNA heteroduplexes with target gene sequences.^[^ ^21^, ^22^, ^23^ ^]^ In our study, we specifically showed that HOXA‐AS2 interacts with cccDNA through the formation of an RNA‐DNA triplex, rather than an RNA‐DNA heteroduplex. The triplex‐forming oligonucleotide (TFO) site within the lncRNA sequence is rich in GA sequences, which enables the formation of a triplex structure with the target gene by forming Hoogsteen bonds between the RNA and DNA strands.^[^ ^21^, ^22^, ^23^ ^]^ Importantly, we found that deletion of the TFO sequence in HOXA‐AS2 significantly attenuated its ability to repress HBV transcription, indicating that the TFO sequence may be crucial for binding to cccDNA. Furthermore, we employed FISH and subcellular fractionation assays to determine the localization of HOXA‐AS2. Our findings revealed that HOXA‐AS2 is predominantly localized in the nucleus. This is consistent with previous studies demonstrating that nuclear lncRNAs are capable of generating RNA‐DNA triplex structures, which in turn recruit chromatin‐modifying enzymes to specific genomic loci.^[^ ^21^, ^22^, ^23^ ^]^ Consistently with these observations, previous studies have also reported that HOXA‐AS2 recruits the EZH2/PRC2 complex to downstream target genes and inhibits gene transcription by altering the epigenetic state of the gene promoter region.^[^ ^37^, ^38^, ^39^, ^40^ ^]^ Our research also uncovered a significant enrichment of lncRNA HOXA‐AS2 in histone modification‐related biological processes. Through the use of mass spectrometry and bioinformatics analysis, we identified 39 nuclear proteins that specifically bind to HOXA‐AS2. Gene Ontology (GO) analysis of these proteins revealed a significant enrichment in histone deacetylation. Among the biological process of histone deacetylation, MTA1, HDAC1, and HDAC2 showed the highest binding abundance and are known to form deacetylase complexes with transcriptional inhibition activity.^[^ ^27^ ^]^ In our study, we demonstrated that HOXA‐AS2 binds to the MTA1‐HDAC1/2 deacetylase complex. By utilizing RNA interference‐based approaches, we discovered that HOXA‐AS2 primarily interacts with MTA1, rather than HDAC1/2. The flat stem‐loop structure of lncRNA serves as a scaffold, allowing it to bind to proteins and assemble chromatin modification complexes.^[^ ^41^, ^42^ ^]^ Truncation analysis revealed that the 182–392 nt segment of HOXA‐AS2, which forms a flat stem‐loop structure, is essential for its interaction with MTA1. Additionally, previous studies have identified the BAH domain on the N‐terminal portion of MTA1 as the primary RNA‐binding motif.^[^ ^28^ ^]^ Further truncation analysis confirmed that the BAH domain, encompassing amino acids 1‐164 of MTA1, is mainly responsible for the interaction between HOXA‐AS2 and MTA1. These findings suggest that MTA1 mediates HOXA‐AS2 recruitment of HDAC1/2 complexes. Prior research indicates that HDAC1 recruitment can lead to a decrease in the acetylation modification level of cccDNA, thereby inhibiting HBV replication.^[^ ^36^ ^]^ ChIP experiments revealed that HOXA‐AS2 recruits the MTA1‐HDAC1/2 complex to cccDNA, with MTA1 playing a mediating role, and HDAC1/2 exerting a deacetylation effect. This provides further evidence of how HDAC1/2 are specifically recruited to cccDNA.

In our study, we delved into the mechanism behind HBV‐induced upregulation of HOXA‐AS2. We observed an intriguing pattern wherein HOXA‐AS2 expression was not activated during low levels of HBV replication but could be induced during high levels of replication. This phenomenon may be explained by the ability of viral proteins to regulate the expression of host factors.^[^ ^18^, ^32^, ^33^ ^]^ In our experiments, we demonstrated that HBV DNA polymerase (HBV DNA pol) played a significant role in enhancing HOXA‐AS2 expression by upregulating the expression of the transcription factor AP‐2*α*. This finding indicates that HBV DNA pol‐mediated upregulation of HOXA‐AS2 occurs through the activation of AP‐2*α*. Moreover, we discovered that HOXA‐AS2 binds to cccDNA and recruits the MTA1‐HDAC1/2 deacetylase complex to inhibit cccDNA transcription, resulting in reduced HBV replication. These findings suggest that HOXA‐AS2 serves as a novel host restriction factor that helps maintain the balance of cccDNA transcription. This concept aligns with previous studies that propose the existence of a self‐restricting mechanism within hepatocytes, which prevents excessive activation of HBV.^[^ ^43^, ^44^ ^]^ Additionally, HBV infection is known to induce oxidative stress and cause cellular damage. Our study revealed that upregulation of HOXA‐AS2 contributes to alleviating HBV‐induced oxidative stress and oxidative damage. This suggests that HOXA‐AS2 plays a protective role in HBV‐infected cells.

**Figure 9:**
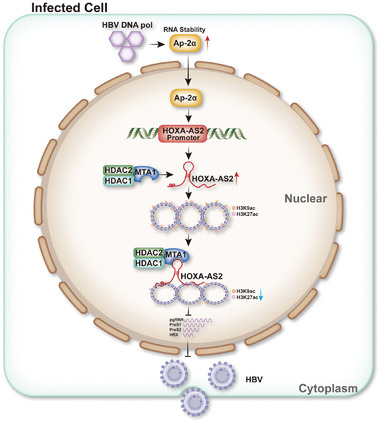
Detailed flow diagram of the regulation process of HOXA‐AS2 on HBV replication through a HBV DNA pol‐AP‐2*α*‐HOXA‐AS2‐MTA1‐HDAC1/2 complex axle. In this study, HBV DNA pol induces HOXA‐AS2 through the transcription factor AP‐2*α* after establishing persistent high‐level HBV replication. Subsequently, HOXA‐AS2 diminishes HBV replication by directly binding to cccDNA and acting as a scaffold to recruit the MTA1‐HDAC1/2 complex. This leads to reduced acetylation and transcriptional activity of cccDNA. These findings provide new insights into the epigenetic inhibition of cccDNA transcription by lncRNAs and the prevention of HBV overactivation.

In summary, our study has uncovered a novel mechanism to self‐limit HBV replication. We have demonstrated that HBV DNA pol upregulates the expression of HOXA‐AS2 after establishing persistent high‐level HBV replication. Subsequently, HOXA‐AS2 reduces HBV replication by directly binding to cccDNA and acting as a scaffold to recruit the MTA1‐HDAC1/2 complex. This results in decreased acetylation and transcriptional activity of cccDNA (**Figure** **9**). These findings provide new insights into the epigenetic inhibition of cccDNA transcription by lncRNAs and the prevention of HBV overactivation.

## 4 Experimental Section

### Cell Culture

HepG2 cells and Hepa 1–6 cells were obtained from the American Type Culture Collection (ATCC). HepG2‐NTCP cells and HepAD38 cells were generously provided by Prof. Ningshao Xia from Xiamen University, Fujian, China. Primary human hepatocytes (PHH) were purchased from Liver Biotechnology (Shenzhen) Co., Ltd. HepG2 cell lines were cultured in Dulbecco's modified Eagle medium (DMEM) (D6429, Sigma–Aldrich, USA) supplemented with 10% fetal bovine serum (FBS) (10270, Corning, New York, USA), 100 U mL^−1^ penicillin, and 100 µg mL^−1^ streptomycin (SV30010, HyClone, USA). Hepa 1–6 cell lines cells were cultured in DMEM (1.5 g L^−1^ NaHCO_3_) (iCell‐128‐0001, iCell, China) supplemented with 10% FBS, 100 U mL^−1^ penicillin, and 100 µg mL^−1^ streptomycin. HepG2‐NTCP cells were cultured in DMEM with 10% FBS, 100 U mL^−1^ penicillin, and 100 µg mL^−1^ streptomycin in the presence of 2 µg mL^−1^ Doxycycline (ID0670, Solarbio, China). HepAD38 cells were cultured in DMEM with 10% FBS and 400 µg mL^−1^ of G418 (345810, Merck Millipore, Germany). Primary human hepatocytes (PHH) were maintained in a PHH maintenance medium (LV‐WEM001, Liver Biotechnology (Shenzhen) Co., Ltd, China). All cell cultures were incubated in a humidified atmosphere at 37 °C with 5% CO_2_.

### Patients

Liver biopsies were obtained from five HBeAg‐positive chronic hepatitis B (CHB) patients and five normal liver tissues from patients with biliary‐related diseases (Table S2, Supporting Information). The liver tissue samples were stored at −80 °C prior to use. The study protocol was approved by the Ethical Committee of the Chongqing Medical University (Approval Number: 2023046), and written informed consent was obtained from all patients, in accordance with the Declaration of Helsinki.

### Animal Experimentation

All animal experiments in this study were conducted in accordance with the guidelines outlined in the “Guide for the Care and Use of Laboratory Animals” and approved by the Animal Ethics Committee of Chongqing Medical University (Approval Number: IACUC‐CQMU‐2023‐07034).

### Plasmids and Antibodies

Lentivirus expressing HOXA‐AS2 was purchased from Shanghai Genechem Company Limited (lentivector CMV‐MCS‐EF1‐ZsGreen1‐T2A‐puromycin). For inhibition of HOXA‐AS2 in cells, LNA Gapmers against human HOXA‐AS2 were purchased from QIAGEN (Cat#339511). HOXA‐AS2 specific Gapmers and scrambled control LNA Gapmers were delivered to cells by Lipofectamine 3000 Transfection Reagent at a final concentration of 50 nmol L^−1^. The primers and siRNAs used in the study are listed in Table S1, Supporting Information. Plasmids expressing MTA1, HDAC1, HDAC2, and HOXA‐AS2 were constructed by amplifying the coding sequences (CDS) of MTA1, HDAC1, HDAC2, and the cDNA of HOXA‐AS2 using high fidelity polymerase (R045, Takara Bio, Japan) and cloning them into the pcDNA3.1 vector. pCH9/3091 was presented by Prof. Lin Lan (The Third Military Medical University, China). pCH9/3091 containing a 1.1‐unit length HBV genome‐wide (1818‐3182(0)−1988 of HBV) (Genotype D, GenBank accession No. V01460) driven by the CMV promoter for HBV pregenomic RNA (pgRNA) transcription. The siRNAs and plasmids were transfected into cells using Lipofectamine 3000 Transfection Reagent (L3000015, ThermoFisher, USA).

Mouse anti‐flag monoclonal antibody (F1804) was obtained from Sigma–Aldrich (USA). Mouse anti‐GAPDH monoclonal antibody (sc‐47724) and rabbit anti‐*β*‐actin monoclonal antibody (sc‐47778) were obtained from Santa Cruz Biotechnology (USA). Rabbit anti‐H3K9ac polyclonal antibody (17‐658), rabbit anti‐H3 acetylation (Ac‐H3) polyclonal antibody (06‐599), mouse anti‐RNA polymerase II (Pol II) monoclonal antibody (17‐620), normal rabbit IgG (NI01), and normal mouse IgG (12‐371) were obtained from Merck Millipore (Germany). Rabbit anti‐MTA1 monoclonal antibody (#5647), mouse anti‐HDAC1 monoclonal antibody (#5356), mouse anti‐HDAC2 monoclonal antibody (#5113), rabbit anti‐His‐Tag monoclonal antibody (#12698), rabbit anti‐H3K27ac monoclonal antibody (#8173) and mouse anti‐Histone H3 monoclonal antibody (#3638) were obtained from Cell Signaling Technology (USA). Rabbit anti‐MTA1 monoclonal antibody (ab288765) and rabbit anti‐AP‐2‐alpha monoclonal antibody (ab108311) were obtained from Abcam (Britain). Mouse anti‐HBc was kindly provided by prof. Xuefei Cai (Chongqing Medical University, Chongqing, China).

### Virus Particles Production and Cell Infection

For virus production, the HepAD38 cells was cultured in DMEM supplemented with 10% FBS and 400 µg mL^−1^ G418. The HBV virions were concentrated from the clarified supernatant through overnight precipitation in 5% polyethylene glycol (PEG)8000. The mixture was then centrifuged at 4 °C to obtain a centrifugal sediment. The sediment was re‐dissolved in serum‐free Opti‐MEM at a volume that was 1% of the original supernatant samples. For infection, the cells were seeded in plates and then inoculated with 500 viral genome equivalents (vge) of HBV particles per cell. The HBV particles were diluted in a culture medium supplemented with 2% dimethyl sulfoxide (DMSO) and 4% PEG8000. After 24 h post‐infection, the cells were washed three times with phosphate buffer saline (PBS) and cultured in a maintenance medium containing 2.5% DMSO. The culture medium was changed every 2 days until cell harvest.

The HBV DNA pol‐deficient viral particles were constructed by co‐transfecting the pCH9/3091‐Δpol plasmid and pcDNA3.1‐pol plasmid into Huh‐7 cells. The pCH9/3091‐Δpol plasmid carries 1.1 copies of the HBV genome with a start codon mutation in the HBV DNA pol gene. The pcDNA3.1‐pol plasmid expresses the HBV DNA polymerase protein. The virus concentration and infection experiments were consistent with wild‐type HBV particles.

### Real‐Time Reverse‐Transcription PCR

Total RNA was extracted from the cells using TRNzol Universal reagent (DP424, TIANGEN, China), followed by DNase treatment to remove any contaminating genomic DNA. For reverse transcription, 1 µg of DNase‐treated RNA was used with the IScript cDNA Synthesis kit (KR116‐02, TIANGEN, China) to generate cDNA. A Fast Start Universal SYBR Green Master was used for real‐time PCR analysis. The relative quantification of target genes was performed using the 2^−ΔΔCt^ method. The endogenous control *β*‐actin was used for normalization. All primers used in the study are listed in Table S1, Supporting Information.

### Hirt Extraction of HBV cccDNA and Analysis

To extract the covalently closed circular DNA (cccDNA) of HBV, the cells were lysed in 500 µL SDS lysis buffer (50 mmol L^−1^ Tris‐HCl, pH8.0, 10 mmol L^−1^ EDTA, 150 mmol L^−1^ NaCl, 1% SDS) at 37 °C for 20 min. Next, 125 µL of 2.5 m KCl was added to the lysate, and the mixture was left to rest overnight at 4 °C. After centrifugation at 12 000 g for 20 min, the supernatant containing the cccDNA was collected. The viral DNA was then isolated by phenol/chloroform (1:1) extraction, and ethanol precipitation. The extracted DNA was subsequently digested with exonuclease V (M0345S, New England Biolabs, USA) for 30 min at 37 °C to remove linear genomic DNA and HBV replication intermediates. The cccDNA was amplified using Taq‐man probe PCR. The specific primers and probe used for cccDNA amplification are listed in Table S1, Supporting Information.

### HBV Core DNA Extraction and Quantification

For the extraction of HBV core DNA, the cells were lysed in 500 µL lysis buffer (10 mmol L^−1^ Tris‐HCl pH8.0, 1 mmol L^−1^ EDTA, 1% NP‐40, 2% sucrose) and incubated for 15 min at 37 °C. The nuclei were removed by centrifugation at 16 000 g for 5 min. 10 µL of the cytoplasmic cell lysate was put aside and *β*‐actin was detected by western blot. 40 IU mL^−1^ DNase I and 10 mmol L^−1^ MgCl_2_ were added for 4 h at 37 °C to remove free DNA. The HBV core capsids were precipitated using 5% PEG8000, and then incubated with proteinase K overnight at 45 °C to release the HBV DNA. The viral DNA was isolated by phenol/chloroform (1:1) extraction, followed by ethanol precipitation. The quantification of HBV DNA was performed using absolute quantification PCR with the specific primers listed in Table S1, Supporting Information.

### Southern Blot

Intracellular HBV DNA samples were subjected to 1% agarose gel electrophoresis and then transferred onto a pretreated nylon membrane following the instructions of the DIG‐High Prime DNA Labelling and Detection Starter Kit (11585614910, Roche, Germany). The membrane was crosslinked with UV light and prehybridized. Next, a digoxigenin‐labeled HBV full‐length genomic DNA probe was added to the membrane and hybridized overnight at 42 °C. After hybridization, the membrane was incubated in blocking solution for 30 min and then in antibody solution for another 30 min at 37 °C. Finally, the signal was visualized by exposing the membrane to an X‐ray film.

### Northern Blot

Total RNA from HBV‐infected cells was extracted and electrophoresed on 1.4% formaldehyde‐agarose gel. The RNA samples were transferred onto a pretreated nylon membrane following the instructions of the DIG Northern Starter Kit (12039672910, Roche, Germany). The membrane was UV‐crosslinked and prehybridized. The membrane was then hybridized with a digoxigenin‐labeled specific RNA probe (corresponding to nucleotides 126 to 1225 of the HBV genome) overnight at 68 °C. The membrane was incubated in blocking solution for 30 min and antibody solution for 30 min at 37 °C. The signal was detected by exposing it to an X‐ray film. The primers used to amplify the probe of HBV RNA are listed in Table S1, Supporting Information.

The experimental procedure for HOXA‐AS2 Northern blot was consistent with the HBV RNA Northern blot detection method, with the difference being the cell sources, which were HBV‐infected or uninfected cells. The probe was a digoxigenin‐labeled specific RNA probe designed to target nucleotides 54 to 492 of HOXA‐AS2. The probe sequence for HOXA‐AS2 as well as the primers used to amplify the probe are listed in Table S1, Supporting Information.

### Chromatin Immunoprecipitation

Chromatin immunoprecipitation (ChIP) assays were performed using the Magna ChIP A/G Chromatin Immunoprecipitation Kit (17‐10086, Merck Millipore, Germany) and with minor modifications.^[^ ^45^ ^]^ HBV‐infected cells were resuspended in 500 µL of ChIP Cell Lysis Buffer and incubated for 15 min at 4 °C. The lysate was then centrifuged to pellet the nuclei. The nuclei were fixed in 1% formaldehyde for 30 min at 4 °C to cross‐link the DNA‐protein complexes. Cross‐linked nuclei were resuspended in a 500 µL of Nuclear Lysis Buffer. The chromatin solution was sonicated to generate 200 to 1000 bp DNA fragments. After centrifugation, the chromatin supernatant was diluted 1:10 in a dilution buffer. The diluted chromatin was subjected to immunoprecipitation overnight at 4 °C using specific antibodies and 20 µL of protein A/G magnetic beads (17‐10086, Merck Millipore, Germany). Immunoprecipitation with normal IgG antibodies was conducted in the experiment to exclude nonspecific binding. After the reverse cross‐linking, immunoprecipitated chromatin was purified by phenol/chloroform (1:1) extraction and ethanol precipitation and analyzed by Taq‐man probe PCR using a specific cccDNA primer and probe. Host genes glyceraldehyde‐3‐phosphate dehydrogenase (GAPDH) and cardiac gene myosin heavy chain 6 (MYH6) were used as control genes. The primers and probe used are listed in Table S1, Supporting Information.

The experimental procedure for HOXA‐AS2 promoter ChIP detection was similar to the HBV cccDNA ChIP detection method, and the primers used are listed in Table S1, Supporting Information.

### Immunofluorescence Staining

Frozen sections of mice liver tissue or cells grown on coverslips were fixed in 4% paraformaldehyde for 15 min and then permeabilized with 0.5% Triton X‐100 for 30 min at room temperature. The cells were subsequently incubated with blocking buffer (2% bovine serum albumin (BSA) in PBS) for 1 h at room temperature and then stained with the indicated primary antibodies overnight at 4 °C. After washing with PBS, bound antibodies were detected using appropriate secondary antibodies (Alexa Fluor 488 (goat anti‐rabbit, 1:1000), Alexa Fluor 488 (goat anti‐mouse, 1:1000), Alexa Fluor 555 (goat anti‐rabbit, 1:1000)). Nuclei were stained with 4′,6‐diamidino‐2‐phenylindole (DAPI). Images were captured by a confocal laser scanning microscope (LEICA DMi8, Germany).

### RNA Immunoprecipitation (RIP)

Whole‐cell extracts were prepared in lysis buffer (300 mmol L^−1^ NaCl, 50 mmol L^−1^ Tris‐HCl pH 7.4, 0.5% NP‐40, and 0.1% DEPC) with protease inhibitor and RNase inhibitor on ice for 30 min, followed by centrifugation at 15 000 g for 20 min at 4 °C. The indicated antibody was added to the clarified lysate and hybridized on the rotator overnight at 4 °C. ChIP‐Grade Protein A/G Magnetic Beads (16‐663, Sigma–Aldrich, USA) were added to the reaction and hybridized at room temperature for 2 h. The precipitated RNA was washed four times using washing buffer (300 mmol L^−1^ NaCl, 50 mmol L^−1^ Tris‐HCl pH 7.4, 0.5% NP‐40, and 0.1% DEPC). After washing, the retrieved RNA was isolated using TRNzol Universal reagent and subjected to real‐time PCR analysis. Total RNA (input controls) and negative IgG controls were detected simultaneously. Specific primers for RIP assay were provided in Table S1, Supporting Information.

### RNA Pull‐Down Assay

The sense and antisense transcripts of HOXA‐AS2 were transcribed and biotinylated using the Ribo RNAmax‐T7 Kit (C11001, RIBOBIO, China) and biotin RNA labeling mix (20163, Thermo Scientific) according to the manufacturer's protocol. Then, 100 pmol of biotinylated RNA was chemically coupled to streptavidin‐linked magnetic beads (20164, Thermo Scientific) at room temperature for 2 h. Add 200 µg of total protein extracted from HepG2‐NTCP cells to the RNA‐loaded beads and incubated overnight at 4 °C. After washing, the precipitated proteins were eluted in a 1×SDS loading Buffer and separated by SDS‐PAGE, followed by silver staining or proceed to mass spectrometry. The retrieved proteins were detected by western blot. To identify potential HOXA‐AS2‐interacted proteins, the number of unique peptides of detected proteins should be equal to or greater than 2 (Cut‐off = unique peptide ≥ 2). Then, through the Venn analysis, the HOXA‐AS2 antisense transcript‐interacted proteins were excluded from the HOXA‐AS2‐sense group, and the nuclear‐localized proteins were selected as potential candidates for further analysis. The primers of HOXA‐AS2 used for in vitro transcription were listed in Table S1, Supporting Information. The antibodies used for RNA pull‐down were listed in Experimental Section.

### Chromatin Isolation by RNA Purification (ChIRP)

ChIRP assays were performed as previously described.^[^ ^46^ ^]^ Antisense DNA probes were designed against HOXA‐AS2 using an online designer at https://www.biosearchtech.com/support/tools/designsoftware/chirp‐probe‐designer, biotin‐TEG‐labeled at its 3′ terminus and divided into odd or even groups. Six days after infection with HBV, HepG2‐NTCP nuclear pellets were crosslinked in 1% glutaraldehyde (Cat#G5882, Sigma–Aldrich, USA) at room temperature for 10 min, and quenched with 125 mmol L^−1^ glycine for 5 min. The pellets were washed in PBS, frozen in liquid nitrogen, and stored at −80 °C. Pellets were resuspended in ChIRP lysis buffer and sheared to 100–500 bp fragments at 4 °C. Cleared lysates were hybridized with probes at 37 °C for 4 h on a rotator followed by incubation with magnetic streptavidin beads for another 30 min at 37 °C with shaking. After washing and elution, RNA and DNA were purified accordingly. Real‐time PCR was performed as described above. The HOXA‐AS2‐bound DNA sample was then used for sequencing. The probes used in the ChIRP assay are listed in Table S1, Supporting Information.

### In Vitro Triplex Pull‐Down Assay

100 fmol of exonuclease I‐digested PCR‐fragments (HBV genome (∆LBR1/2/3/4/5) fragment or LBR1 fragment (425/458) of HBV genome) were incubated with 1 pmol of biotin‐labeled HOXA‐AS2 in 10 mmol L^−1^ Tris‐HCl (pH 7.5), 20 mmol L^−1^ KCl, 10 mmol L^−1^ MgCl_2_, 0.05% Tween 20, and 100 U of RNase inhibitor (Thermo, USA) for 20 min at room temperature. RNA‐DNA complexes were bound to streptavidin‐coated Dynabeads (Life Technologies, USA), washed three times with a buffer containing 150 mmol L^−1^ KCl, 10 mmol L^−1^ Tris‐HCl (pH 7.5), 5 mmol L^−1^ MgCl_2_, 0.5% NP‐40, and 100 U of RNase inhibitor and once with buffer containing 15 mmol L^−1^ KCl, 10 mmol L^−1^ Tris‐HCl (pH 7.5), and 5 mmol L^−1^ MgCl_2_. The RNA‐associated DNA was eluted in 1% SDS, 50 mmol L^−1^ Tris‐HCl (pH 8.0), and 10 mmol L^−1^ EDTA for 5 min at 65 °C and digested with RNase A (50 ng mL^−1^) for 30 min at 37 °C and proteinase K (200 ng mL^−1^) for 15 min at 37 °C. The recovered DNA was subjected to real‐time PCR using primers specific for the HBV fragment (Table S1, Supporting Information).

### Electrophoretic Mobility Shift Assay (EMSA)

One picomole of biotin‐labeled double‐stranded oligonucleotide comprising LBR1 fragment (425/458) of HBV genome was incubated with an 80‐fold molar excess of in vitro transcribed HOXA‐AS2 (1/139) for 1 h at 37 °C in 40 mmol L^−1^ Tris‐acetate (pH 7.5), 20 mmol L^−1^ KCl, 10 mmol L^−1^ Mg(CH_3_COO)_2_, and 10% glycerol. Triplex formation was monitored by EMSA on 12% polyacrylamide gels containing 40 mmol L^−1^ Tris‐acetate (pH 7.5) and 10 mmol L^−1^ MgCl_2_, and the gel contents were transferred to a nylon membrane. The biotin‐labeled LBR1 fragment of HBV genome and its complexes were visualized using the LightShift Chemiluminescent EMSA kit (20148, Thermo, USA). The probes and primers used in the EMSA assay are listed in Table S1, Supporting Information.

### RNA Fluorescence In Situ Hybridization (RNA‐FISH)

RNA‐FISH assay was performed using Stellaris RNA FISH Buffers (Stellaris, LGC Biosciences) according to the manual. HOXA‐AS2 expression in cells was measured by RNA‐FISH and the Quasar570 (Q570)‐conjugated Stellaris FISH probes are listed in Table S1, Supporting Information (Stellaris, LGC Biosciences). Briefly, frozen sections of mice liver tissue or cells grown on coverslips were fixed in 4% paraformaldehyde for 10 min and then incubated with 250 µL RNA probes in the dark overnight at 37 °C. After being washed by Buffer A three times, the frozen sections or coverslips were incubated with DAPI for nuclear counterstaining, and images were captured by using a confocal laser scanning microscope (LEICA DMi8, LEICA, Weztlar, Germany).

### Nuclear and Cytoplasm RNA Extraction

To explore the sub‐cellular distribution of HOXA‐AS2, RNA in cytoplasmic and nuclear fractions was isolated by using the cytoplasmic & nuclear RNA purification kit (21000, Norgen, CA) according to the manufacturer's instructions. GAPDH was used as the endogenous cytoplasmic control and U6 was used as the endogenous nuclear control. The relative distribution of HOXA‐AS2 was detected by real‐time PCR.

### The Nascent RNA Capture Assay

HBV‐infected HepG2‐NTCP cells were incubated with 0.2 mmol L^−1^ 5‐ethynyl uridine (EU) for 24 h before harvest. Total cellular RNA was extracted by using TRNzol. The newly synthesized EU‐labeled RNA was conjugated to biotin and purified from the total RNA by the Click‐iT Nascent RNA Capture Kit (Cat# C10365, Invitrogen, USA) according to the manufacturer's protocols. EU‐labeled HBV 3.5‐kb RNA were quantified by real‐time PCR.

### Determination of Intracellular Reactive Oxygen Species (ROS)

ROS have been detected with 2′,7′‐dichlorofluorescein diacetate (DCFH‐DA, S0033S, Beyotime) according to the manufacturers manuals. ROS were measured based on the intracellular peroxide‐dependent oxidation of DCFH‐DA to form the fluorescent compound 2′,7′‐dichlorofluorescein (DCF). HepG2‐NTCP cells were incubated with 10 µmol L^−1^ DCFH‐DA for 30 min at 37 °C. Then, the cells were washed with PBS for three times, 200 µL of PBS was added to each well, and fluorescence intensity was determined with a fluorescence microscopy or flow cytometer. The fluorescence of 10 000 cells was collected for each of the three independent experiments.

### Comet Assay

The alkaline comet assay (WLA123a, Wanleibio, China) for assessing DNA damage in HepG2‐NTCP cells was performed according to the manufacturer's protocol. DNA damage was assessed through the evaluation of tail moments using ImageJ software (version 1.52; National Institutes of Health, Bethesda, MD) with the OpenComet plugin.^[^ ^47^ ^]^

### Determination of GSH/GSSG

The glutathione (GSH) and oxidized glutathione (GSSG) in HepG2‐NTCP cells were quantified using GSH/GSSG‐Glo Assay (V6611, Promega, USA) according to the optimized protocol provided in the kit.

### Apurinic/Apyrimidinic (AP) Sites Assay

Genomic DNA of HepG2‐NTCP cells was extracted with Wizard Genomic DNA Purification Kit (A1120, Promega, USA). AP sites in the genomic DNA were measured by DNA Damage Quantification Kit (DK02, DOJINDO, Japan) according to the manufacturer's protocol. Briefly, the genomic DNA was labeled by Aldehyde Reactive Probe (ARP). Then standard ARP DNA and purified ARP‐treated sample DNA were fixed on the 96 well plates with DNA Binding Solution. The number of AP sites in the sample DNA was determined by the biotin‐avidin‐peroxidase assay.

### Cell Counting Kit‐8 Cell Viability Assay

Cell viability was evaluated using Cell Counting Kit‐8 (HY‐K0301, MedChemExpress, USA). The cells were treated with CCK‐8 reagent and incubated for 2 h at 37 °C. The amount of formazan generated by cellular mitochondrial dehydrogenase activity was measured at 450 nm by using Synergy H1 (BioTek Instruments, Inc). The difference in cell viability was detected by comparing the absorbance of each well. All experiments were repeated in triplicate.

### Enzyme‐Linked Immunosorbent Assay

HBeAg and HBsAg in the cell culture supernatants were determined by enzyme‐linked immunosorbent assay (ELISA) according to the manufacturer's protocol (Kehua Bio‐Engineering Co., Shanghai, China).

### Statistical Analysis

All statistical analyses were performed using Prism software package version 8 (GraphPad Software, San Diego, CA, USA). Mann–Whitney U test was applied to compare the statistical significance of differences between groups. All data were presented as mean ± standard deviation (SD) of three representative experiment. All statistical tests were two‐tail; p value of less than 0.05 was considered statistically significant.

## Conflict of Interest

The authors declare no conflict of interest.

## Author Contributions

Y.Q., J.R., H.Y., X.H. contributed equally to this work. J.C. designed the research; Y.Q., J.R., H.Y., X.H., S.C., W.C., Z.Y., F.S., C.W., S.Y., P.C., D.W., F.R., and A.H. performed experiments and the acquisition of data. Y.Q., J.R., H.Y., and X.H. analyzed and interpreted data. J.C., Y.Q., J.R., and H.Y. wrote the paper and critically reviewed the manuscript. All authors read and approved the final version of the manuscript.

## Supporting information

Supporting Information

## Data Availability

The data that support the findings of this study are available from the corresponding author upon reasonable request.
