# Supplementary material for: HOXA‐AS2 Epigenetically Inhibits HBV Transcription by Recruiting the MTA1‐HDAC1/2 Deacetylase Complex to cccDNA Minichromosome
Source: Adv Sci (Weinh). 2024 Apr 22;11(24):2306810. doi: 10.1002/advs.202306810 (PMC11200093; doi:10.1002/advs.202306810)
Supplement: Supplementary file 1 — Supporting Information [file ADVS-11-2306810-s001.pdf]

ADVANCED SCIENCE
Open Access

Supporting Information

for *Adv. Sci.*, DOI 10.1002/advs.202306810

HOXA-AS2 Epigenetically Inhibits HBV Transcription by Recruiting the MTA1-HDAC1/2 Deacetylase Complex to cccDNA Minichromosome

*YiPing Qin, JiHua Ren, HaiBo Yu, Xin He, ShengTao Cheng, WeiXian Chen, Zhen Yang, FengMing Sun, ChunDuo Wang, SiYu Yuan, Peng Chen, DaiQing Wu, Fang Ren, AiLong Huang and Juan Chen**

Figure S1-9
Table S1-2

Supporting Information

HOXA-AS2 Epigenetically Inhibits HBV Transcription by Recruiting the MTA1-HDAC1/2 Deacetylase Complex to cccDNA Minichromosome

*YiPing Qin, JiHua Ren, HaiBo Yu, Xin He, ShengTao Cheng, WeiXian Chen, Zhen Yang, FengMing Sun, ChunDuo Wang, SiYu Yuan, Peng Chen, DaiQing Wu, Fang Ren, AiLong Huang, Juan Chen**

Y. Qin, J. Ren, H. Yu, X. He, S. Cheng, W. Chen, Z. Yang, F. Sun, C. Wang, S. Yuan, P. Chen, D. Wu, F. Ren, A. Huang, J. Chen
Institute for Viral Hepatitis, Key Laboratory of Molecular Biology for Infectious Diseases (Ministry of Education), Department of Infectious Diseases, The Second Affiliated Hospital, Chongqing Medical University, Chongqing 400010, China
Email: chenjuan2014@cqmu.edu.cn
Y. Qin
Chongqing Key Laboratory of Translational Research for Cancer Metastasis and Individualized Treatment, Chongqing University Cancer Hospital, Chongqing 400030, China
J. Chen
State Key Laboratory of Ultrasound in Medicine and Engineering, College of Biomedical Engineering, Chongqing Medical University, Chongqing 400016, China
F. Sun
Key Laboratory of Clinical Laboratory Diagnostics (Ministry of Education), College of Laboratory Medicine, Chongqing Medical University, Chongqing, 400016, China

Figure S1

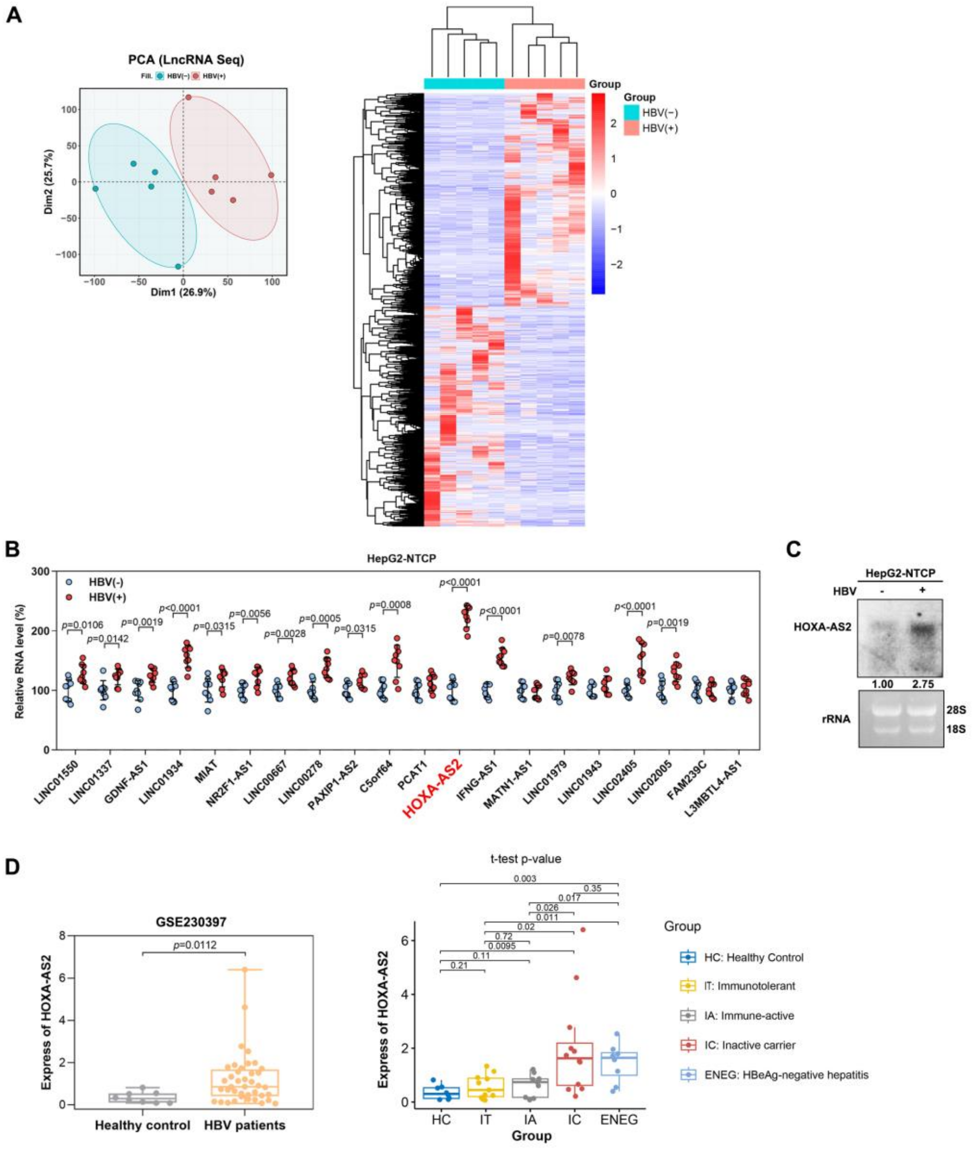

Figure S1. Screening and identification of lncRNAs associated with HBV replication. (A) LncRNA sequencing was performed in 5 pairs of HBV-positive liver tissues and HBV-negative liver tissues. Principal-component analysis (PCA) of differentially expressed lncRNAs was shown (left). Each dot represents one sample. Blue; HBV-negative liver tissue samples. Red; HBV-positive liver tissue samples. Heatmap of expression data for differentially expressed lncRNAs was shown (right). Upregulated and downregulated lncRNAs were colored in red and blue, respectively. (B) The expression levels of these up-regulated lncRNAs in HBV-infected HepG2-NTCP cells and uninfected cells were detected by real-time PCR. (C) The expression level of HOXA-AS2 in HBV-infected HepG2-NTCP cells and uninfected cells was analysed by northern blot. Ribosomal RNAs (28S and 18S) serves as loading controls. (D) Left: Comparison of HOXA-AS2 levels between healthy controls (n = 8)

and all HBV patients (n = 40). Right: Analysis of HOXA-AS2 levels in healthy controls (n = 8) and patients at different stages of HBV infection (IT: n = 11, IA: n = 9, IC: n = 12, ENEG: n = 8) (GSE230397).

Figure S2

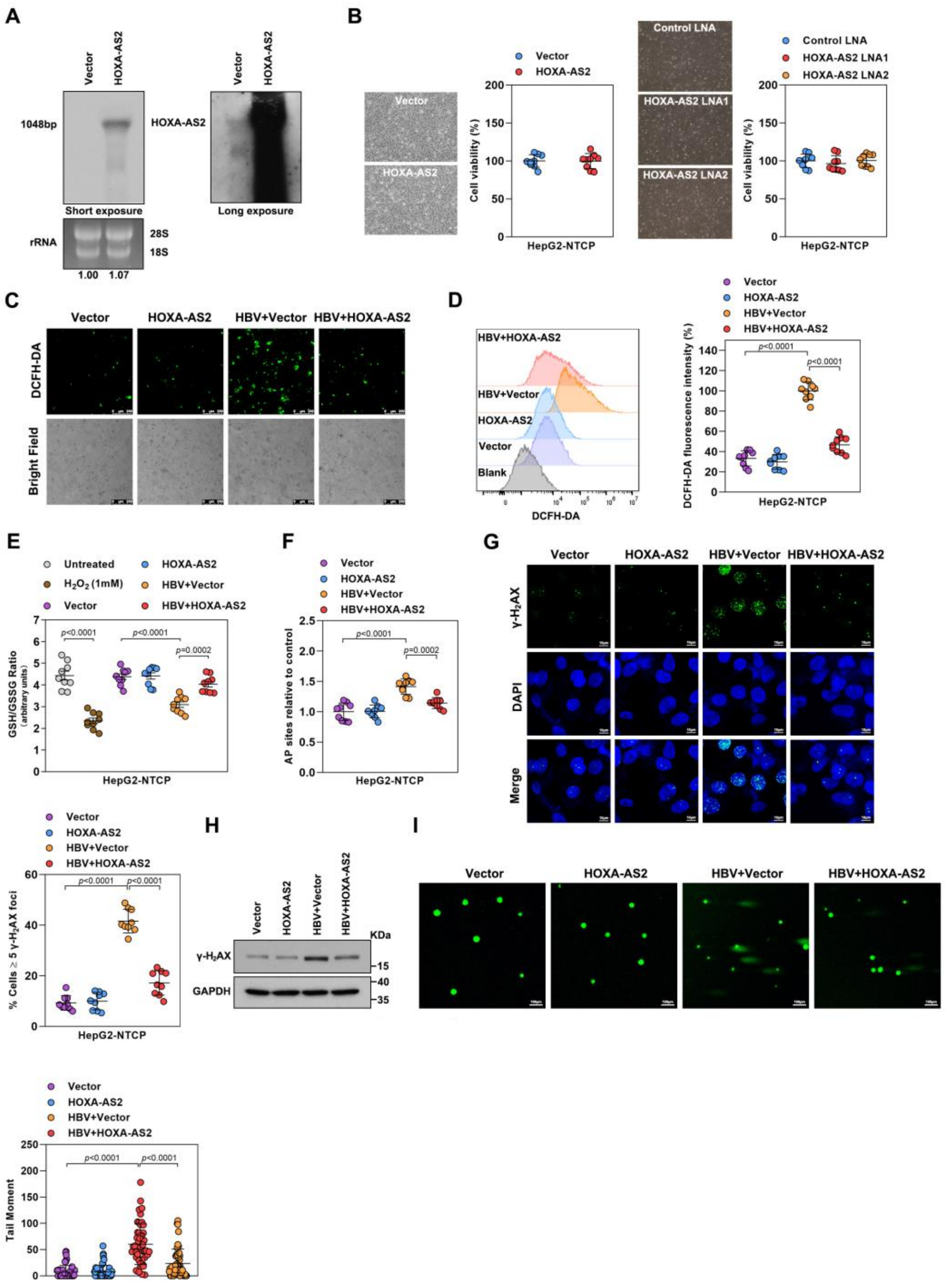

Figure S2. HOXA-AS2 reduces HBV-induced oxidative damage. (A) After 24 h of HBV inoculation in HepG2-NTCP cells, the cells were transduced with lentivirus expressing vector or HOXA-AS2 for 5 days. The expression level of HOXA-AS2 was

analysed by northern blot. Ribosomal RNAs (28S and 18S) served as loading controls. (B) HBV-infected HepG2-NTCP cells were transduced with lentivirus expressing vector or HOXA-AS2, or transfected with control locked nucleic acid (LNA) or HOXA-AS2 LNA for 5 days. The cell viability was investigated by fluorescence microscope and cell counting kit-8 assay. (C-I) HepG2-NTCP cells and HBV-infected HepG2-NTCP cells were transduced with lentivirus expressing vector or HOXA-AS2 for 5 days. The levels of reactive oxygen species (ROS) in the cells were visualized using a fluorescent probe, 2',7'-dichlorodihydrofluorescein diacetate (DCFH-DA), and analyzed by fluorescence microscopy (C) and flow cytometry (D). Representative images are shown. Scale bar, 250 µm. Quantification of fluorescence intensity was performed by flow cytometry. (E) The glutathione (GSH)/oxidized glutathione (GSSG) ratio in the cells was measured using the GSH/GSSG-Glo™ Assay. The H_2O_2 treatment group was used as a positive control. (F) The number of apurinic/apyrimidinic (AP) sites, an indicator of DNA damage, was estimated using a DNA Damage Quantification Kit. (G) Histone H_2AX phosphorylation (γ-H_2AX), another marker of DNA damage, was visualized in HepG2-NTCP cells by immunofluorescence staining. Quantification of γ-H_2AX foci was performed. Representative images are shown. Scale bar, 10 µm. (H) Intracellular γ-H_2AX levels were also measured by western blot. GAPDH was used as a loading control. (I) DNA damage in HepG2-NTCP cells was detected using the comet assay. The tail moments, indicative of DNA damage, were quantified. Representative images are shown. Scale bar, 100 µm. Data are shown as the median and interquartile range of the tail moments in three independent experiments (n = 49 [HepG2-NTCP were transduced with Vector], n = 50 [HepG2-NTCP were transduced with HOXA-AS2], n = 48 [HBV-infected HepG2-NTCP were transduced with Vector], and n = 49 [HBV-infected HepG2-NTCP were transduced with HOXA-AS2] cells). For (B), (D-G) and (I) representative data from at least 3 independent experiments are shown. Data are presented as mean ± SD. Statistical analysis was performed using the Mann-Whitney U test.

Figure S3

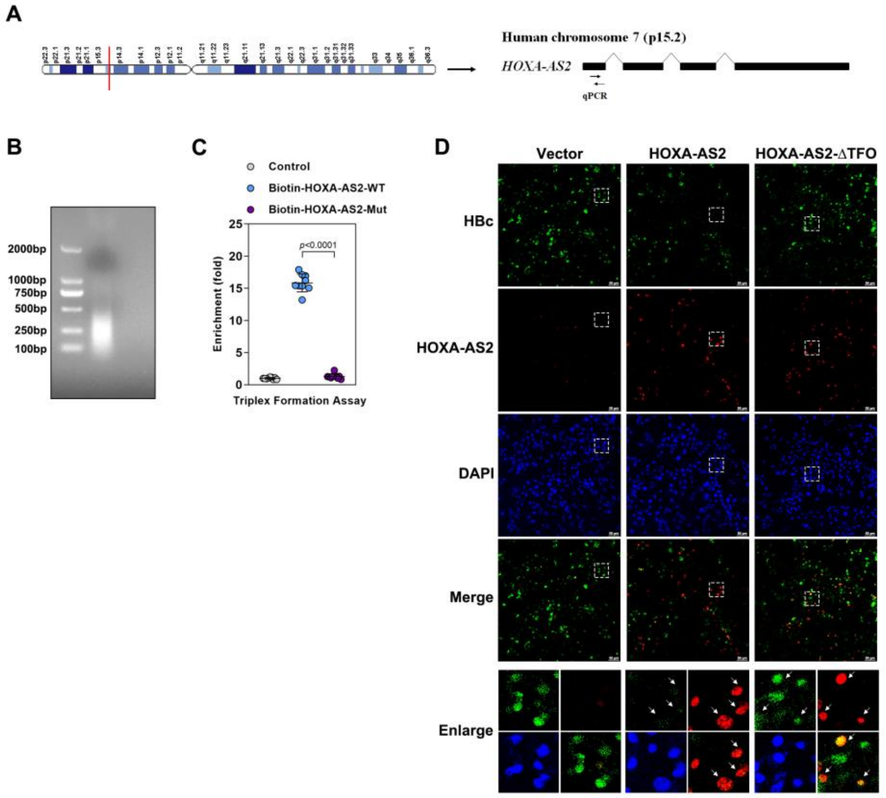

Figure S3. HOXA-AS2 formed an RNA-DNA triplex with cccDNA. (A) Schematic representation of exons and transcripts of HOXA-AS2 and its loci on human chromosome 7p15.2 in UCSC Genome browser (GRCh38.p13). (B) In the ChIRP assay, HBV-infected HepG2-NTCP nuclear pellets lysate was sheared by ultrasound. The product of ultrasonication was analyzed by gel electrophoresis and the result showed nuclear lysate was sheared to 100-500 bp fragments. (C) Reactions containing biotin-labeled HOXA-AS2 WT (1/139) or Mut (deletion of the TFO region (45/68) in HOXA-AS2 WT) and a 40-molar excess of HBV DNA were treated with 0.5 U RNase H or 0.5 ng RNase A for 30 min at room temperature. Upon binding to the streptavidin beads, the associated HBV DNA was analysed by real-time PCR. Normalized data were shown as relative fold enrichment to the control group. (D) HBV-infected HepG2-NTCP cells were transfected with the HOXA-AS2 plasmid or its TFO mutant for 5 days. HOXA-AS2 was visualized by RNA-FISH (red), and immunofluorescence staining of HBc (green) in HBV-infected HepG2-NTCP cells was performed. Nuclei were stained with DAPI (blue). Scale bar, 50 μm. FISH stands for fluorescence in situ hybridization.

Figure S4

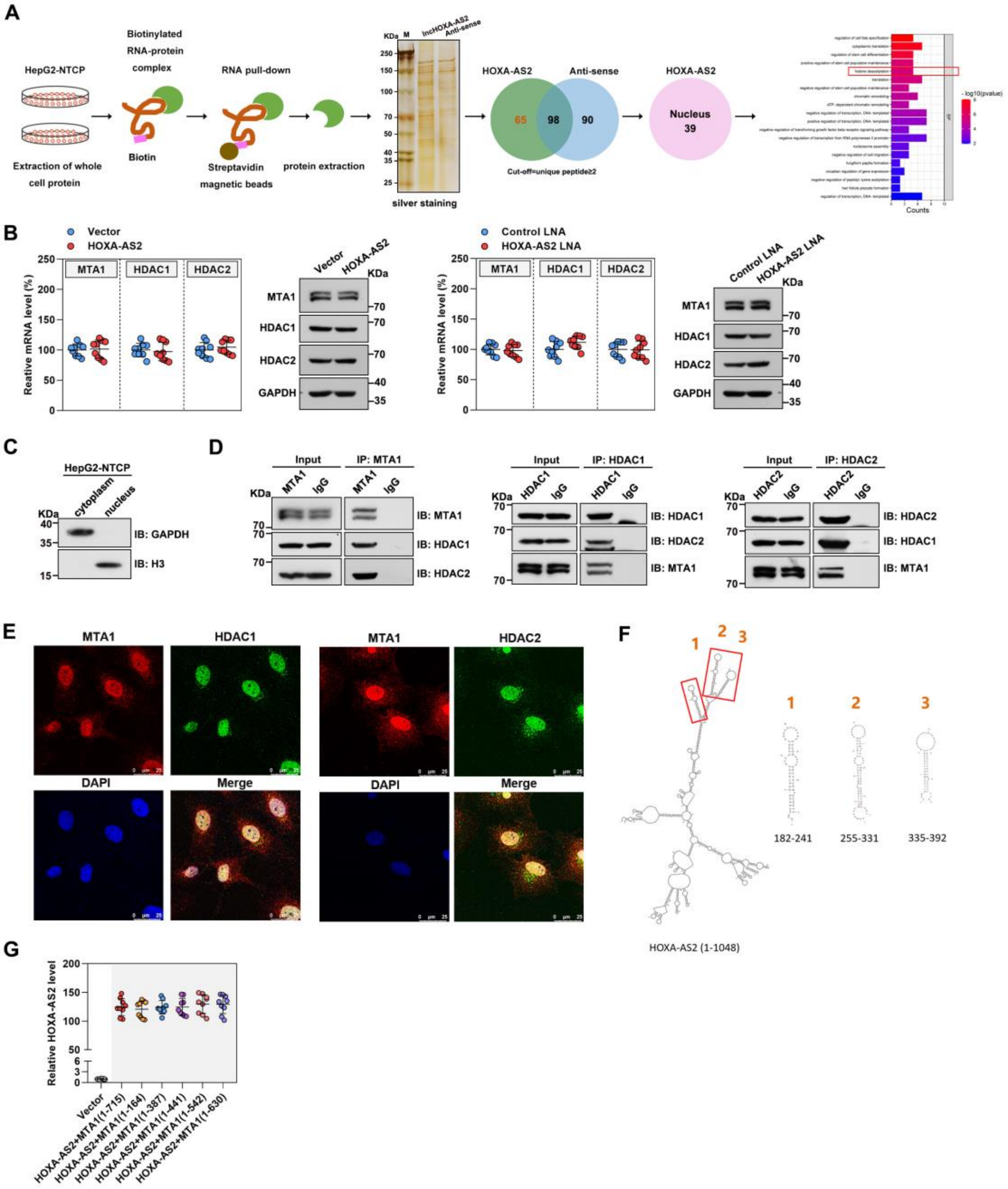

Figure S4. HOXA-AS2 binding MTA1-HDAC1/2 complex. (A) The specific associations of proteins with biotinylated-HOXA-AS2 were detected by streptavidin RNA-pull down assay and further analyzed by sliver staining and mass spectrometry. The HOXA-AS2 specific binding nucleoproteins were analyzed by Gene Ontology (GO) enrichment analysis. (B) After 24 h of HBV inoculation in HepG2-NTCP cells, the cells were transduced with lentivirus expressing vector or HOXA-AS2, or transfected with control locked nucleic acid (LNA) or HOXA-AS2 LNA for 5 days. The expression levels of MTA1, HDAC1 and HDAC2 were detected using real-time PCR and western blot. (C) GAPDH and H3 were used as a marker for cytoplasmic and nuclear fractions, respectively. (D-E) The interaction between MTA1, HDAC1 and

HDAC2 was detected by Co-IP (D) and immunofluorescence assay (E). (F) Predicted secondary structures of HOXA-AS2 and its flat stem-loop structure were presented. (G) HepG2-NTCP cells were transfected with HOXA-AS2 and plasmids expressing different His-tagged-MTA1 truncates. The expression level of HOXA-AS2 was analysed by real-time PCR.

Figure S5

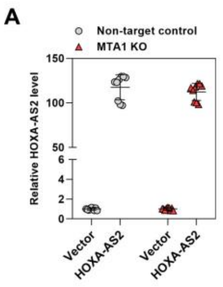

Figure S5. Overexpression efficiency of HOXA-AS2. (A) HepG2-NTCP cells with MTA1 knockout were infected with HBV for 24 h and then transduced with lentivirus expressing vector or HOXA-AS2 for 5 days. The expression level of HOXA-AS2 was detected by real-time PCR.

Figure S6

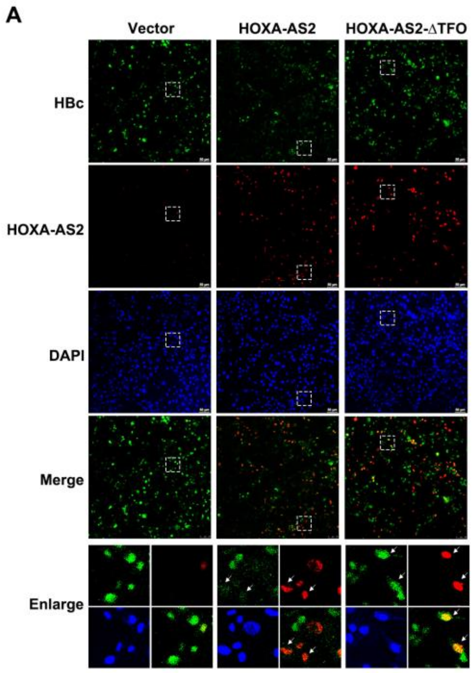

Figure S6. HOXA-AS2 forms an RNA-DNA triplex with cccDNA to inhibit HBV replication. (A) HBV-infected HepG2-NTCP cells were transfected with the HOXA-AS2 plasmid or its TFO mutant for 5 days. HOXA-AS2 was visualized by RNA-FISH (red), and immunofluorescence staining of HBc (green) in HBV-infected HepG2-NTCP cells was performed. Nuclei were stained with DAPI (blue). Scale bar, 50 µm.

Figure S7

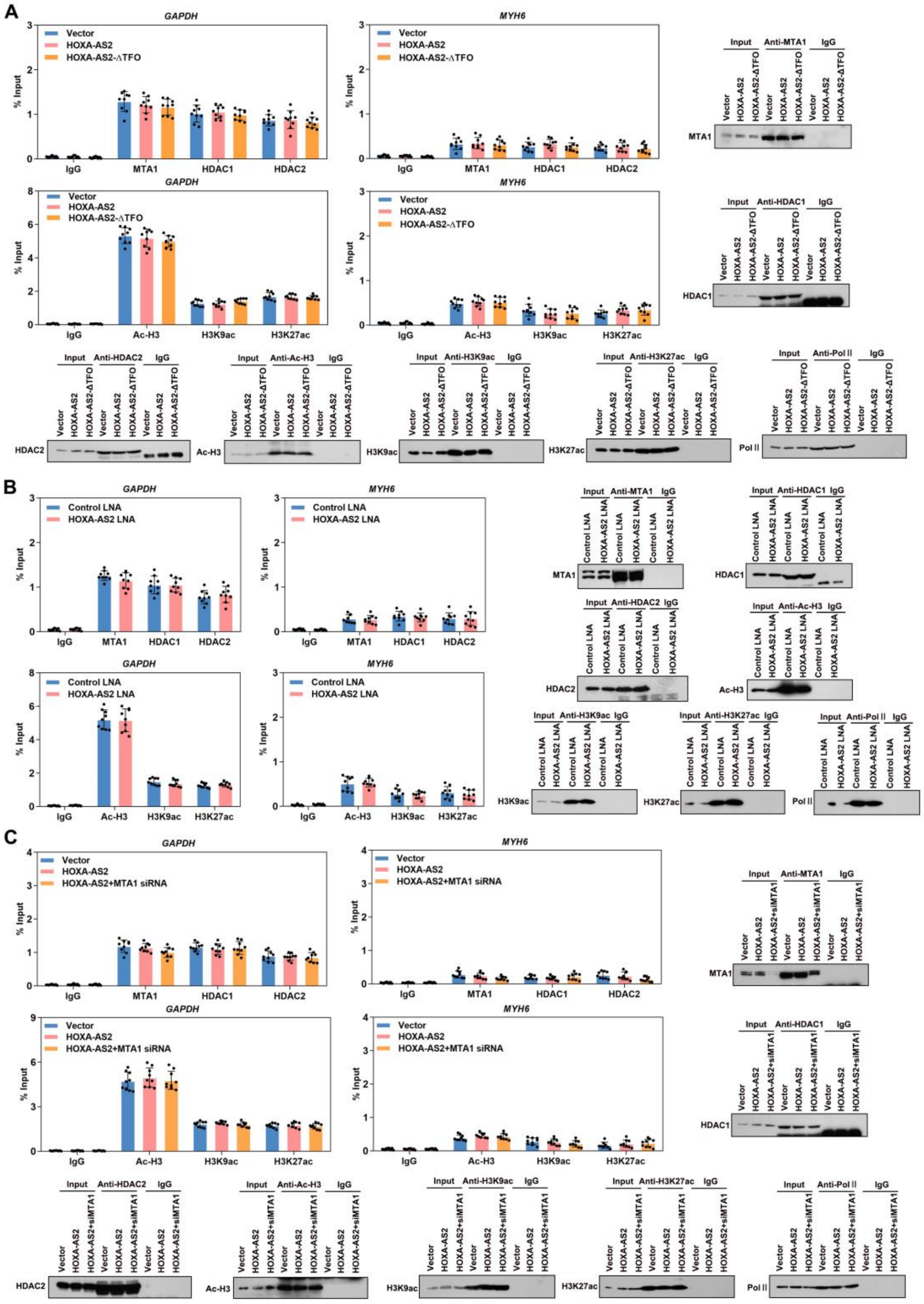

Figure S7. HOXA-AS2 does not affect the occupancy of the MTA1-HDAC1/2 complex on GAPDH and MYH6 promoters. (A) HBV-infected HepG2-NTCP cells were transfected with HOXA-AS2/HOXA-AS2-ΔTFO plasmid for 5 days. (B) HBV-infected HepG2-NTCP cells were transfected with control LNA or HOXA-AS2 LNA for 5 days. (C) HBV-infected HepG2-NTCP cells were transfected with HOXA-AS2 plasmid and MTA1 siRNA for 5 days. (A-C) The levels of MTA1,

HDAC1, HDAC2, Ac-H3, H3K9ac and H3K27ac associated with GAPDH or MYH6 promoter were analysed by ChIP assay with the indicated antibodies. Western blotting analysis were performed to confirm that MTA1, HDAC1, HDAC2, Ac-H3, H3K9ac and H3K27ac were immunoprecipitated in the ChIP experiments.

Figure S8

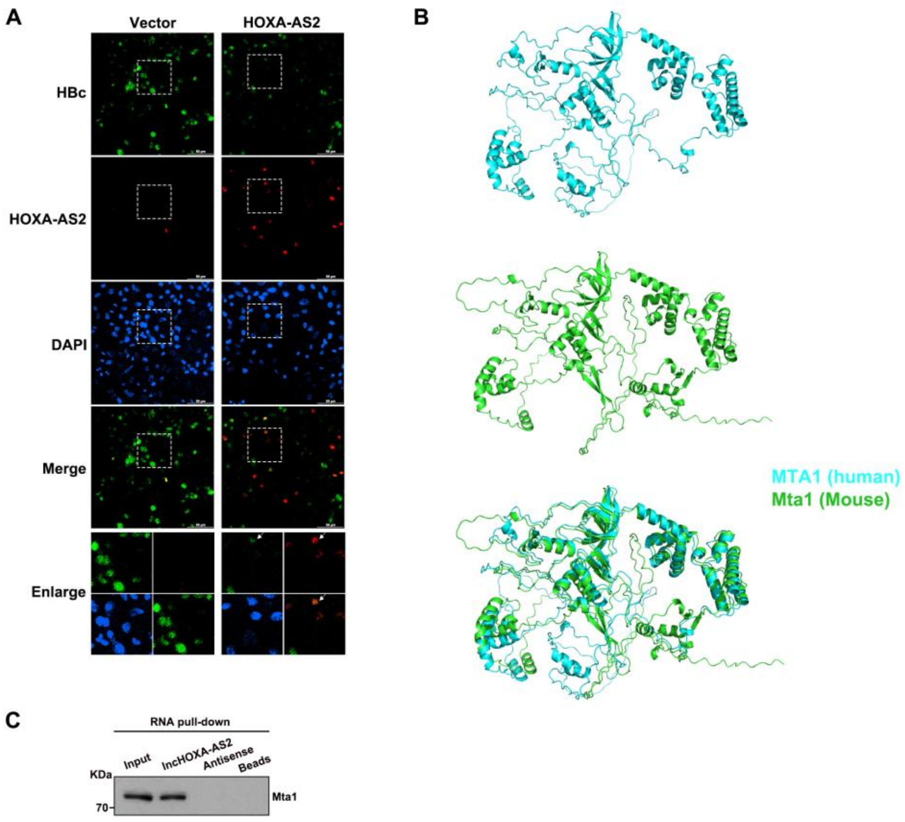

Figure S8. HOXA-AS2 inhibits cccDNA transcription *in vivo.* (A) C57BL/6 mice were injected with prcccDNA and pCMV-Cre. After one week, the mice were injected with a lentivirus-packaged vector or HOXA-AS2 for 21 days. HOXA-AS2 was visualized by RNA-FISH (red), and immunofluorescence staining of HBc (green) in mouse liver was performed. Nuclei were stained with DAPI (blue). Scale bar, 50 µm. (B) The structure prediction and alignment of human MTA1 and mouse Mta1 were performed using AlphaFold2. (C) RNA pull-down and western blot were performed to detect the specific associations of HOXA-AS2 with mouse Mta1.

Figure S9

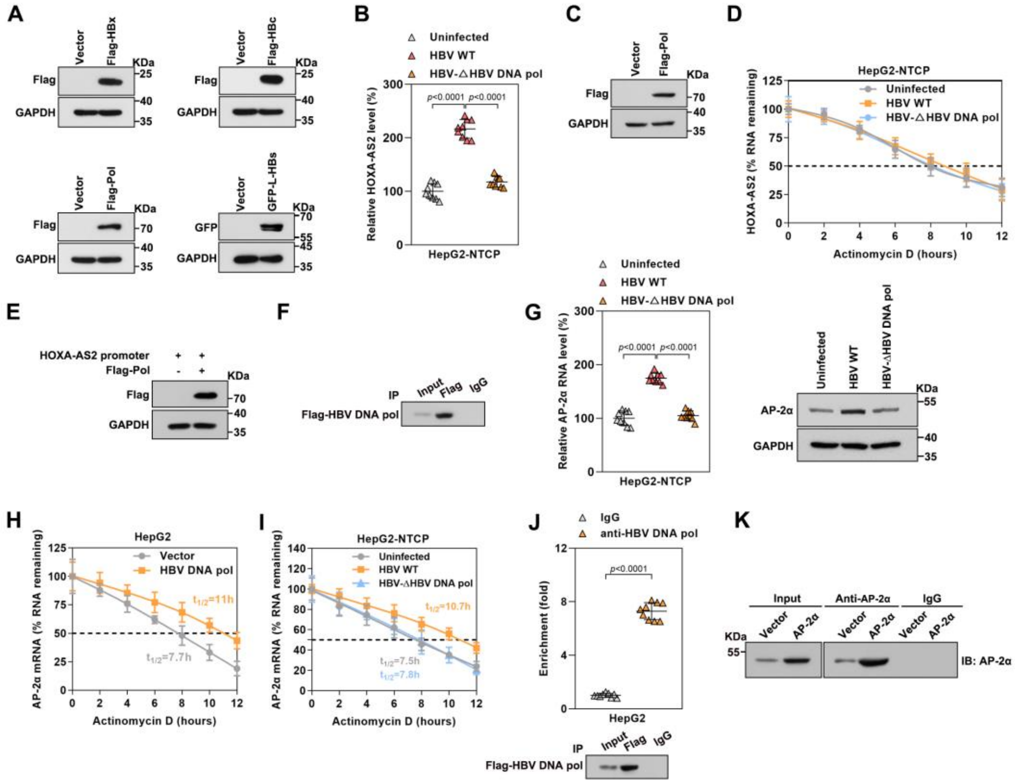

Figure S9. HBV DNA pol induces HOXA-AS2 expression via AP-2α. (A) HepG2 cells were transfected with different HBV protein expression plasmids for 3 days, and the expression levels of HBx, HBc, HBV DNA pol and L-HBs were analysed by western blot. (B) HepG2-NTCP cells were infected with wild-type HBV particles (HBV WT) or HBV DNA pol-deficient viral particles (HBV-ΔHBV DNA pol) for 9 days, and the level of HOXA-AS2 was measured using real-time PCR. (C) HepG2 cells were transfected with plasmid expressing Flag-HBV DNA pol for 3 days, and the level of Flag-HBV DNA pol was detected by western blot. (D) HepG2-NTCP cells were infected with wild-type HBV particles (HBV WT) or HBV DNA pol-deficient viral particles (HBV-ΔHBV DNA pol) for 9 days and incubated with 5 µg mL^{-1} actinomycin D for the indicated times. The half-life of HOXA-AS2 was detected by real-time PCR. (E) HepG2 cells were transfected with HOXA-AS2 promoter and Flag-HBV DNA pol expression plasmid, and the level of Flag-HBV DNA pol was detected by western blot. (F) HepG2 cells were transfected with HOXA-AS2 promoter and Flag-HBV DNA pol expression plasmid, and the enrichment of HBV DNA pol on the HOXA-AS2 promoter was determined by ChIP assay. Western blotting analysis were performed to confirm that Flag-HBV DNA pol was immunoprecipitated in the ChIP experiments. (G) HepG2-NTCP cells were infected with wild-type HBV particles (HBV WT) or HBV DNA pol-deficient viral particles (HBV-ΔHBV DNA pol) for 9 days, and the level of the transcription factor AP-2α was detected by real-time PCR and western blot. (H) HepG2 cells were transfected with plasmid expressing HBV DNA pol for 3 days and

incubated with 5 μg mL^{-1} actinomycin D for the indicated times. The stability of AP-2α was detected by real-time PCR. (I) HepG2-NTCP cells were infected with wild-type HBV particles (HBV WT) or HBV DNA pol-deficient viral particles (HBV-ΔHBV DNA pol) for 9 days and incubated with 5 μg mL^{-1} actinomycin D for the indicated times. The half-life of AP-2α was detected by real-time PCR. (J) RIP assay for the in vivo interaction between AP-2α and Flag-HBV DNA pol. HepG2 cells were transfected with plasmid expressing Flag-HBV DNA pol. RIP assay was performed using anti-Flag antibody at day 3 post transfection. Western blotting analysis were performed to confirm that Flag-HBV DNA pol was immunoprecipitated in the RIP experiments. (K) HepG2 cells were co-transfected with AP-2α expression plasmid and HOXA-AS2 promoter plasmid, and the level of AP-2α associated with HOXA-AS2 promoter was examined by ChIP assay. Western blotting analysis was performed to confirm that AP-2α was immunoprecipitated in the ChIP experiments.

Table S1. Primers and siRNA in this study

Gene and primer	Primer sequence (5' to 3')
HBV cccDNA-F	GTGCACTTCGCTTCACCTCT
HBV cccDNA-R	AGCTTGGAGGCTTGAACAGT
HBV cccDNA probe	ACGTCGCATGGAGACCACCGTGAACGCC
HBV 3.5-kb RNA-F	GCCTTAGAGTCTCCTGAGCA
HBV 3.5-kb RNA-R	GAGGGAGTTCTTCTTCTAGG
Total HBV RNA-F	ACCGACCTTGAGGCATACTT
Total HBV RNA-R	GCCTACAGCCTCCTAGTACA
β-actin-F	CTCTTCCAGCCTTCCTTCCT
β-actin-R	AGCACTGTGTTGGCGTACAG
GAPDH-F	ACCACAGTCCATGCCATCAC
GAPDH-R	TCCACCACCCTGTTGCTGTA
U6-F	CTCGCTTCGGCAGCACA
U6-R	AACGCTTCACGAATTTGCGT
HBV DNA-F	CCTAGTAGTCAGTTATGTCAAC
HBV DNA-R	TCTATAAGCTGGAGGAGTGCGA
GAPDH-ChIP-F	TACTAGCGGTTTTACGGGCG
GAPDH-ChIP-R	TCGAA AGGAGGAGCAGAGAGCGA
MYH6-ChIP-F	AGAAGCTGCGCTCAGACCTGTCTCG
MYH6-ChIP-R	TCCAGGTCCCGCCGCATCTT
HOXA-AS2-ChIP-F	CTTCACCGGCCTCCAACTTG
HOXA-AS2-ChIP-R	AACGGTGTGTACCCTTGGTT
HOXA-AS2-F	CCCGTAGGAAGAACCGATGA
HOXA-AS2-R	TTTAGGCCTTCGCAGACAGC
MTA1-F	ACGCAACCCTGTCAGTCTG
MTA1-R	GGGCAGGTCCACCATTTCC
HDAC1-F	CCGCATGACTCATAATTTGCTG

HDAC1-R	ATTGGCTTTGTGAGGGCGATA
HDAC2-F	TCCGCATGACCCATAACTTGC
HDAC2-R	CCGCCAGTTGAGAGCTGAC
GR-α-F	TGCCGCTATCGAAAATGTCTT
GR-α-R	GGGTAGGGGTGAGTTGTGGT
AP-2α-F	AGGTCAATCTCCCTACACGAG
AP-2α-R	GGAGTAAGGATCTTGCGACTGG
C/EBPβ-F	CTTCAGCCCGTACCTGGAG
C/EBPβ-R	GGAGAGGAAGTCGTGGTGC
YY1-F	ACGGCTTCGAGGATCAGATTC
YY1-R	TGACCAGCGTTTGTTCAATGT
STAT4-F	GCTTAACAGCCTCGATTTCAAGA
STAT4-R	GAGCATGGTGTTCATTAACAGGT
c-Myc-F	GTCAAGAGGCGAACACACAAC
c-Myc-R	TTGGACGGACAGGATGTATGC
NF-AT1-F	CGATTCGGAGAGCCGGATAG
NF-AT1-R	TGGGACGGAGTGATCTCGAT
FOXP3-F	GTGGCCCGGATGTGAGAAG
FOXP3-R	GGAGCCCTTGTCGGATGATG
TFIID-F	GAGCCAAGAGTGAAGAACAGTC
TFIID-R	GCTCCCCACCATATTCTGAATCT
HNF-3α-F	GCAATACTCGCCTTACGGCT
HNF-3α-R	TACACACCTTGGTAGTACGCC
NF-1-F	AGATGAAACGATGCTGGTCAAA
NF-1-R	CCTGTAACCTGGTAGAAATGCGA
TFII-I-F	TCGGATGAGTGTAGATGCTGT
TFII-I-R	CCACTGTGGATTTGCCTAAAGC
ER-α-F	GAAAGGTGGGATACGAAAAGACC
ER-α-R	GCTGTTCTTCTTAGAGCGTTTGA
GCF-F	CGGACGAAGGCTCAGAATCC
GCF-R	CCAAGAGAGCTAGAACTGTCAGA
IRF-2-F	CATGCGGCTAGACATGGGTG
IRF-2-R	GCTTTCCTGTATGGATTGCCC
LINC01550-F	AGTGCCCTCAATACGAAG
LINC01550-R	AACCTCAGAGCCCACCAC
LINC01337-F	ACTAGAAAAGCAATGACTAAGGGT
LINC01337-R	TCTGTGGCTCAAGATGCTGG
GDNF-AS1-F	AATTCCCAGCCTTCTGTGCA
GDNF-AS1-R	AGACCCGGATTCCAGTCTCA
LINC01934-F	ATCAGGGCAGAGCTGAGAGA
LINC01934-R	CCACAAAAAGCACCTGGAGC
MIAT-F	ATTTCCTCTGGTGCATGGGG
MIAT-R	CAAAAGAGCCAACCACACCG
NR2F1-AS1-F	TATCTGCGCACTGAGACGTC

NR2F1-AS1-R	GCAGCTTACATCACGGCATG
LINC00667-F	GTGGGTAGGAAACAGTCGGG
LINC00667-R	AGCTCCCTAGGTGATGCTGA
LINC00278-F	CGACAGGTTTGCTGTCTCCT
LINC00278-R	CGCCACTGGGAATCAAGCTT
PAXIP1-AS2-F	TTCAAACCAGTCGCTCTGCT
PAXIP1-AS2-R	TCACATTCAGCGTTCGGTCA
C5orf64-F	TTGCCTAACAGGAACCCACC
C5orf64-R	AGCAGGGTCCAGTGTTCATG
PCAT1-F	AAACCGCAACATCACCAACG
PCAT1-R	TTCACCGTGTTAGCCAGGACA
IFNG-AS1-F	ACAGCTGATGATGGTGGCAA
IFNG-AS1-R	GCCTTTTCCTAGTCCGGCTT
MATN1-AS1-F	AGAACTCCTGCTTCACGGTG
MATN1-AS1-R	TCCTTCTGGCTCATACCGGA
LINC01979-F	CTGGTCACATGTGCTGGGAT
LINC01979-R	GGCAAACCACAAATCGGCTT
LINC01943-F	CCCCTACTAACAGCCCTCCT
LINC01943-R	GGACCATGTGACCTTGGAGG
LINC02405-F	TTGGCAAGAATCCCTGGGAC
LINC02405-R	GCCTCTTGTCAGGAGCCATT
LINC02005-F	GTTGCTGCCACCACTACAGA
LINC02005-R	AAGGGCGCTGAGTGTTCTAC
FAM239C-F	TCGGAAGAGAAGCACTGCTG
FAM239C-R	GTGTGAGCCACCATGCTTTG
L3MBTL4-AS1-F	GCTCCAAGGACAGTGGGTTT
L3MBTL4-AS1-R	ACCATCTCACACAAGCCAGA
**siRNA**	**siRNA sequence (5’ to 3’)**
AP-2α-siRNA-1sense	GCUCCGGGAUCAGCAACCCUU
AP-2α-siRNA-1antisense	AAGGGUUGCUGAUCCCGGAGC
AP-2α-siRNA-2sense	CCAAUGAGCAAGUGACAAGAA
AP-2α-siRNA-2antisense	UUCUUGUCACUUGCUCAUUGG
MTA1-siRNA-1sense	GCAUCAUUGAGUACUACUATT
MTA1-siRNA-1antisense	UAGUAGUACUCAAUGAUGCTT
MTA1-siRNA-2sense	GCAUCUUGUUGGACAUAUUTT
MTA1-siRNA-2antisense	AAUAUGUCCAACAAGAUGCTT
MTA1-siRNA-3sense	GAUACUCUCCACAAGAACATT
MTA1-siRNA-3antisense	UGUUCUUGUGGAGAGUAUCTT
HDAC2-siRNA-1sense	CAAUAAGACCAGAUAACAUTT
HDAC2-siRNA-1antisense	AUGUUAUCUGGUCUUAUUGTT
HDAC2-siRNA-2sense	CAAUGAGAGAUGGUAUAGATT
HDAC2-siRNA-2antisense	UCUAUACCAUCUCUCAUUGTT
HDAC2-siRNA-3sense	GAUAGCUUGUGAUGAAGAATT
HDAC2-siRNA-3antisense	UUCUUCAUCACAAGCUAUCTT

HDAC1-siRNA-1sense	GCUCCAUCCGUCCAGAUAATT
HDAC1-siRNA-1antisense	UUAUCUGGACGGAUGGAGCTT
HDAC1-siRNA-2sense	GGAGAGUACUUCCCAGGAATT
HDAC1-siRNA-2antisense	UUCCUGGGAAGUACUCUCCTT
HDAC1-siRNA-3sense	GCCGGUCAUGUCCAAAGUATT
HDAC1-siRNA-3antisense	UACUUUGGACAUGACCGGCTT
**RNA-pull down assay primers**	**Primer sequence (5' to 3')**
HOXA-AS2 sense F1	TAATACGACTCACTATAGGGGAAAAGGAAACGCCAAGACA
HOXA-AS2 sense R1	TGGACCTCACAAGATTATTT
HOXA-AS2 antisense F7	TAATACGACTCACTATAGGGTGGACCTCACAAGATTATT
HOXA-AS2 antisense R7	GAAAAGGAAACGCCAAGAC
Truncated-HOXA-AS2 #2 F2	TAATACGACTCACTATAGGGGAAAAGGAAACGCCAAGACA
Truncated-HOXA-AS2 #2 R2	TCTTCGCCGCGTGGGACTTT
Truncated-HOXA-AS2 #3 F3	TAATACGACTCACTATAGGGGAAAAGGAAACGCCAAGACA
Truncated-HOXA-AS2 #3 R3	GGGTCCGGCGAGCGGACGAT
Truncated-HOXA-AS2 #4 F4	TAATACGACTCACTATAGGGGTTTTGGAGCAGCGCTTACC
Truncated-HOXA-AS2 #4 R4	GGGTCCGGCGAGCGGACGAT
Truncated-HOXA-AS2 #5 F5	TAATACGACTCACTATAGGGGTTTTGGAGCAGCGCTTACC
Truncated-HOXA-AS2 #5 R5	TGGACCTCACAAGATTATTT
Truncated-HOXA-AS2 #6 F6	TAATACGACTCACTATAGGGTGGCTTGGAGAAGTTCTGCG
Truncated-HOXA-AS2 #6 R6	TGGACCTCACAAGATTATTT
**CRISPR/Cas9 system (MTA1)**	**sgRNA sequence (5' to 3')**
MTA1-sgRNA 1	CTCTGTGGGCACCTTCGCAC
MTA1-sgRNA 2	CTCCAAGGCCATCTCGGCGC
**RNA-FISH assay (HOXA-AS2)**	**Probe sequence (5' to 3')**
1	CTATGTCTTGGCGTTTCC
2	CATCGGTTCTTCCTACGG
3	TTAGGCCTTCGCAGACAG
4	TCCACGGTTCCTGGCAAC
5	CGGTTGGGAGGAGTTGGC
6	AAAACTCTTCGCCGCGTG
7	TTCTAGGTAAGCGCTGCT
8	AAAGGCGATTCCCTGAGC
9	TATTTGTCTGCAGAGCGC
10	GACCATTTGCTTCTGCTG
11	AAAGCTGACTGCCGGCTC
12	AGCGGAGCGCAGAACTTC
13	CTGTAGACGCAAAGGGCC
14	TAGTCGGGGCAGGGATAG
15	CCTGGGTCTGAGTGAAGG
16	GGCATAGAAACGTGTCCT
17	TCTGAGAGGGCTCCTCTT
18	GAGTAGGAAGGCTCTTGA

19	GCTGAACTCTTGTCAAGC
20	GATTCTGACGGCTCACAT
21	GCTTGAGCTGGATTCACT
22	ATACTTGACCTTGCATGT
23	GTGGCTACATAGAAAGCC
24	AGAGATACTGTTGTCCCC
25	GACCTTGATGGTAGGTGT
**ChIRP assay (HOXA-AS2)**	**Probe sequence (5' to 3')**
1	TTCTTCCTACGGGAAAAGCG
2	TAAGCGCTGCTCCAAAACTC
3	TGCTGTTTATTTGTCTGCAG
4	AGAACTTCTCCAAGCCAGGG
5	AGGCATAGAAACGTGTCCTT
6	CTTGAGCTGAACTCTTGTCA
7	GATACTTGACCTTGCATGTA
8	GTGGCTACATAGAAAGCCAT
9	GATGGTAGGTGTGACATGTT
**LncHOXA-AS2 binding region**	**Sequence (5' to 3')**
LBR1	TGTCTGCGGCGTTTTATCATCTTCCTCTTCATCCTGCTGCTATGCCT CATCTTCTTGTTG
LBR2	TTTGTCTTTGGGTATACATTTAAACCCTAACAAAACAAAGAGATGGG GTTACTCTCTAAATTTTATGGGTTATGTCAT
LBR3	CAATTCTTTGCTGGGGGGAACTAATGACTCTAGCTACCTGGGTGGG TG
LBR4	TGGGCTTTATTCTTCTACTGTACC
LBR5	TCCAGCCTTCAGAGCAAACACCGCAAATCCAGATTGGGACTTCAAT CCCAACAAGGACACCTGGCCAGACGCCAACAAGGTA
**In vitro triplex pull-down assay**	**Primer sequence (5' to 3')**
HBV genome F	ACCGACCTTGAGGCATACTT
HBV genome R	GCCTACAGCCTCCTAGTACA
**EMSA assay**	**Sequence (5' to 3')**
cccDNA LBR1 (425/458) F	GCCTCATCTTCTTGTTGGTTCTTCTGGACTATCA
cccDNA LBR1 (425/458) R	TGATAGTCCAGAAGAACCAACAAGAAGATGAGGC
lncHOXA-AS2 (1/139) F	TAATACGACTCACTATAGGGGAAAAGGAAACGCCAAGACA
lncHOXA-AS2 (1/139) R	TTGGCCCTCCACGGTTCCTG
**Northern blot assay**	**Primer sequence (5' to 3')**
HBV Northern F1	CTCGAGGATTGGGGACCCTG
HBV Northern R1	AATTAACCCTCACTAAAGGGAAGCCCAGGATGATGGGATG
HBV Northern F2	CATCTTCTTGTTGGTTCTTCT
HBV Northern R2	AATTAACCCTCACTAAAGGGATGATGTGTT CTTGTGGCAAG
HBV Northern F3	GCTTTCAGTTATATGGATGATG
HBV Northern R3	AATTAACCCTCACTAAAGGGTGGCCCATGACCAAGCCCCA

HOXA-AS2 Northern F	ATGATGAGCCCTGATGAA
HOXA-AS2 Northern R	TAATACGACTCACTATAGGGCTTCCCTGGGTCTGAGTG
**Northern blot assay**	**Probe sequence (5’ to 3’)**
HOXA-AS2 Northern	ATGATGAGCCCTGATGAAAGAAGGAAGAAGACCCGCTGTCTGCGA<br>AGGCCTAAAGGCCGCGGTTGCCAGGAACCGTGGAGGGCCAACTCC<br>TCCCAACCGCCCTGGTGCAAAGTCCCACGCGGCGAAGAGTTTTGG<br>AGCAGCGCTTACCTAGAAAGATGTTTAAATTCTGAACCAGGAATTG<br>TCTCCAACTCCAGGCGCTCAGGGAATCGCCTTTTCCGGTGTCCAGG<br>CGCTCTGCAGACAAATAAACAGCAGAAGCAAATGGTCACCGAGCC<br>GGCAGTCAGCTTTCTGGGAGTGGGAGATGATGGGGAAAGAGGAAA<br>GAATCGTCCGCTCGCCGGACCCTGGCTTGGAGAAGTTCTGCGCTCC<br>GCTGGGACTCTGCGGGCCCTTTGCGTCTACAGACCTATCCCTGCCC<br>CGACTACCCCTTCACTCAGACCCAGGGAAG

Table S2. Characteristics of studied patients for lncRNA sequencing assay

Table S2. Patient characteristics of liver biopsies included in the lncRNA-sequencing analysis.

Patients	Age (y)	Gender (F/M)	ALT (IU/ml)	HBeAg (IU/ml)	HBsAg (IU/ml)	HBV DNA (IU/ml)
1	36	M	27	-	-	-
2	48	M	17.8	-	-	-
3	32	M	119.7	-	-	-
4	44	M	154	-	-	-
5	30	M	74	-	-	-
6	31	M	125.8	>150	>250	1.41×10^{7}
7	27	M	11.4	>150	>250	8.39×10^{6}
8	33	M	31	>150	>250	1.29×10^{7}
9	44	M	53.1	8.614	70.88	7.70×10^{4}
10	39	M	155	133.495	212.87	3.52×10^{7}

ALT, alanine aminotransferase.
